# Insights into Bacterial Cellulose Biosynthesis from Different Carbon Sources and the Associated Biochemical Transformation Pathways in *Komagataeibacter* sp. W1

**DOI:** 10.3390/polym10090963

**Published:** 2018-08-31

**Authors:** Shan-Shan Wang, Yong-He Han, Jia-Lian Chen, Da-Chun Zhang, Xiao-Xia Shi, Yu-Xuan Ye, Deng-Long Chen, Min Li

**Affiliations:** 1 College of Life Science, Fujian Normal University, Fuzhou 350117, China; sswang1208@163.com; 2 Quangang Petrochemical Research Institute, Fujian Normal University, Quanzhou 362801, China; yhhan@fjnu.edu.cn (Y.-H.H.); zhdchhhu@163.com (D.-C.Z.); 3 College of Environmental Science and Engineering, Fujian Normal University, Fuzhou 350007, China; 18359172332@163.com; 4 State Key Laboratory of Pollution Control and Resource Reuse, School of the Environment, Nanjing University, Nanjing 210023, China; shixx@nju.edu.cn (X.-X.S.); enproye@163.com (Y.-X.Y.); 5 The Innovative Center for Eco-Friendly Polymeric Materials of Fujian Province, Quanzhou 362801, China

**Keywords:** bacterial cellulose, *Komagataeibacter*, carbon sources, genome sequencing, metabolic pathway

## Abstract

Cellulose is the most abundant and widely used biopolymer on earth and can be produced by both plants and micro-organisms. Among bacterial cellulose (BC)-producing bacteria, the strains in genus *Komagataeibacter* have attracted wide attention due to their particular ability in furthering BC production. Our previous study reported a new strain of genus *Komagataeibacter* from a vinegar factory. To evaluate its capacity for BC production from different carbon sources, the present study subjected the strain to media spiked with 2% acetate, ethanol, fructose, glucose, lactose, mannitol or sucrose. Then the BC productivity, BC characteristics and biochemical transformation pathways of various carbon sources were fully investigated. After 14 days of incubation, strain W1 produced 0.040–1.529 g L^−1^ BC, the highest yield being observed in fructose. Unlike BC yields, the morphology and microfibrils of BCs from different carbon sources were similar, with an average diameter of 35–50 nm. X-ray diffraction analysis showed that all membranes produced from various carbon sources had 1–3 typical diffraction peaks, and the highest crystallinity (i.e., 90%) was found for BC produced from mannitol. Similarly, several typical spectra bands obtained by Fourier transform infrared spectroscopy were similar for the BCs produced from different carbon sources, as was the *I_α_* fraction. The genome annotation and Kyoto Encyclopedia of Genes and Genomes analysis revealed that the biochemical transformation pathways associated with the utilization of and BC production from fructose, glucose, glycerol, and mannitol were found in strain W1, but this was not the case for other carbon sources. Our data provides suggestions for further investigations of strain W1 to produce BC by using low molecular weight sugars and gives clues to understand how this strain produces BC based on metabolic pathway analysis.

## 1. Introduction

Cellulose is the most abundant and widely used biopolymer on earth, most of which is produced by plants [1,2]. In addition to plant origin, cellulose is also naturally produced by micro-organisms including bacteria, fungi, and algae [3]. Due to its unique properties such as high purity, high crystallinity, high water-capacity, low production cost, and good biocompatibility, the cellulose produced by bacteria (i.e., bacterial cellulose, BC) has been widely used in the preparation of food hydrocolloids, high-strength recycled paper, cosmetic moisturizer, and medical materials [4,5,6,7]. By an additional modification, BC-based materials can also be used to prepare adsorption or filter membranes for pollutant removal from aqueous solution [8,9] or to prepare electrochemical materials with high-performances [5].

In the presence of glucokinase, phosphoglucomutase and uridine triphosphate (UTP)-glucose-1-phosphate uridylyltransferase, glucose is transformed to glucose-6-phosphate, glucose-1-phosphate, uridine diphosphate (UDP)-glucose, and finally to unbranched β-1,4-d-glucan (i.e., BC) with the aid of cellulose synthases [10,11]. Besides glucose, all substrates which can be transformed to glucose are theoretically available for BC production. For example, four strains of *Komagataeibacter xylinus* studied by Singhsa et al. [12] showed great ability for BC production on using fructose, lactose, maltitol, sucralose, and xylitol. Other substrates such as glycerol, sucrose, and galactose are also good carbon sources for BC production [13,14,15]. Unlike sugars or their derivatives, using ethanol and acetate as the substrates for BC production has been hardly reported [16]. More often, both ethanol and acetate are spiked in media to enhance BC production by improving adenosine triphosphate (ATP) production, which is responsible for energy supply in the tricarboxylic acid (TCA) cycle and sugar metabolisms. Specifically, these processes are achieved by promoting the activities of glucokinase and fructokinase for BC production and inhibiting the activities of gluconokinase and glucose-6-phosphate dehydrogenase in pentose phosphate metabolism for energy production [17,18,19,20,21,22].

Since the carbon sources have different molecular weights, chemical structure, and bioavailability, it can result in varied BC production rates and structural characteristics [12,23]. Among reported carbon sources, glucose is considered the main source for BC production, while fructose produces low yields of BC [14,16]. However, BC production abilities in different bacteria vary significantly, as do the BC properties [12,14,24,25,26]. Moreover, the existing studies mostly focus on the utilization of carbon sources rather than associated transformation mechanisms. Therefore, having an overview of the utilization efficiency of different carbon sources and the associated biochemical transformation pathways in a given BC-producing bacterium can provide good possibilities for optimization and improvement of BC production.

Our previous study reported a BC producer *Komagataeibacter* sp. W1, and the BC morphology, fibril distribution, purity, and functional groups were well characterized [3]. To understand how the strain synthesized BC in the presence of glucose, the draft genome sequence of W1 and the associated genes were also analyzed [3]. However, the BC synthesis using other carbon sources except for glucose is still unknown. In this work, the carbon sources, including acetate, ethanol, fructose, glycerol, lactose, mannitol, and sucrose were used to evaluate their potentials in BC production as compared to glucose. The full aims were to (1) compare BC productivity and characteristics by using different carbon sources; (2) reveal the corresponding biochemical pathways for the transformation of the above carbon sources.

## 2. Materials and Methods 

### 2.1. Micro-Organism, Culture Media and Cultivation

*Komagataeibacter* sp. W1 used in this study was isolated from a vinegar fermentation tank in Quanzhou, China [3].

Hestrin–Schramm (HS) medium was used as the basic medium, which consisted of 2% glucose (C_6_H_8_O_7_·H_2_O), 0.5% yeast extract, 0.5% peptone, 0.68% Na_2_HPO_4_·12H_2_O, 0.115% C_6_H_8_O_7_·H_2_O and 0.051% MgSO_4_·7H_2_O [27]. The medium pH was adjusted to 6.0 using 1.0 M NaOH or HCl.

Before testing the effects of carbon sources on BC production in strain W1, the bacteria were pre-cultured in basic medium at 30 °C for 24 h under agitated condition (180 rpm) and at 30 °C for 7 days under static conditions. After that, the Erlenmeyer flask was vibrated rapidly to separate the bacteria from BC membranes. The established suspension was concentrated at 8000 *g* for 5 min to remove the residual glucose, followed by dilution in sterile Milli-Q to a total volume of 10 mL. Aliquots of re-suspension were transferred to a new 250 mL Erlenmeyer flask, which contained 100 mL HS medium with a final OD_600_ of 0.01. To evaluate the BC productivities in the media spiked with different carbon sources, glucose in HS medium was replaced with acetate (C_2_H_4_O_2_), ethanol (C_2_H_6_O), fructose (C_6_H_12_O_6_), glycerol (C_3_H_8_O_3_), lactose (C_12_H_22_O_11_), mannitol (C_6_H_14_O_6_), and sucrose (C_12_H_22_O_11_), respectively. The structures of various carbon sources used in this study are given in Figure S1. All setups were incubated at 30 °C for 14 days under static conditions.

### 2.2. BC Purification and Yield Calculation

The BC on the HS medium surface was collected and washed with Milli-Q water to remove medium components. To eliminate bacterial cells inside the BC membranes or on the BC surface, the samples were boiled at 100 °C for 2 h in a 0.1 M NaOH bath and anther 2 h in a Milli-Q water bath [3]. The pre-treated BC was soaked in Milli-Q water overnight at room temperature to remove all chemicals. After 24 h of storage at −80 °C, the BC was freeze-dried (FreeZone 6 plus, Labconco, Kansas City, MI, USA) for 24 h [3] and then, weighed.

BC production was recorded as the dry weight within the volume of medium in liter (g L^–1^) [28]. The BC yield was calculated by Equation (1):

(1) $$\mathrm{Yield}\;(\%)=\frac{m_{ce}}{m_{ca}}\times 100$$

where *m_ce_* is dry weight of BC (g) and *m_ca_* is the weight of carbon source spiked in media (g).

### 2.3. BC Characterization

Scanning electron microscopy (SEM, Quanta™ 250 FEG, FEI, Hillsboro, OR, USA), X-ray diffraction (XRD, Bruker D8 ADVANCE, Karlsruhe, Germany) and Fourier transform infrared (FTIR, Thermo Scientific Nicolet iS5, Waltham, MA, USA) spectroscopy were used to characterize BC.

Before analysis, the dried BC was treated by spray-gold and observed by SEM with the spot of 3.0, high voltage of 15 KeV and magnification of 2000×. Subsequently, 100 nanofibrils of the BC were used for diameter calculation by using a Nano Measurer 1.2 (Fudan University, Shanghai, China). In this study, each 10 nm was set as a group and the data were presented as % of total nanofibrils. To have a full prediction of theoretical diameter distribution, the statistical histograms were plotted by using OriginPro 9.0 (OriginLab Corporation, Northampton, MA, USA).

X-ray diffraction was performed by using nickel filtered copper *K*_a_ radiation (*λ* = 0.15406 nm) at a voltage of 40 kV and a filament emission of 30 mA, with 0.1° step, from 4° to 70° (2*θ*, angle). A silicon zero background plate was used to avoid any peak resulting from the sample holder. The established sample holder position and both of the holder and silicon zero background plate were used for XRD analysis [23]. The *d*-spacing between the crystal planes and an apparent crystal size (ACS) approximation were determined using Bragg’s law and Scherrer’s formula by Equations (2) and (3), respectively [2]:

(2) $$d=\frac{\lambda }{2\sin\theta }$$

(3) $$\mathrm{ACS}=\frac{0.9\lambda }{\mathrm{FWHM}\;\cos\theta }$$

where *λ* is the wavelength of the X-rays, *θ* is the angle between the plane and the diffracted or incident beam (i.e., Bragg’s angle), and FWHM is the width of the peak at half the maximum height. FWHM was obtained by Integrated Peaks analysis based on the Peaks and Baseline module in OriginPro 9.0.

Crystallinity index (C.I.) and percentage of crystallinity (% crystalline) of BC were calculated by Equations (4) and (5) [29]:

(4) $$C.I.=\frac{I_{ma}-I_{am}}{I_{ma}}$$

(5) $$\%\;\mathrm{crystalline}=\frac{I_{ma}}{I_{ma}+I_{am}}\times 100$$

where *I_ma_* is the maximum diffraction intensity of the lattice peak between 2*θ* of 22° to 23° and *I_am_* is the minimum diffraction intensity of the amorphous peak (i.e., baseline) between 2*θ* of 18° to 19°.

In addition to SEM and XRD analyses, FTIR was also used to evaluate the BC characteristics based on information of the functional groups and peak annotations. Specifically, attenuated total refection (ATR) mode with 32 scans per measurement and a resolution 0.5 cm^−1^ in the range of 4000–400 cm^−1^ was carried out. Baselines for each sample spectrum were normalized and cellulose *I_α_* content was calculated by Equation (6) [30]:

(6) $${f_{\alpha }}^{\mathrm{IR}}=\frac{A_{\alpha }}{A_{\alpha }+A_{\beta }}$$

where *A_α_* and *A_β_* are the integrated intensities (i.e., the peak heights at 750 and 710 cm^−1^) of the contributions from celluloses *I_α_* and *I_β_*, respectively.

### 2.4. Analysis of Biochemical Transformation Pathways Associated with Carbon Source Metabolisms

To have a full insight into the mechanisms of carbon source transformation and BC biosynthesis, all associated open reading frames (orfs) based on gene prediction and Kyoto Encyclopedia of Genes and Genomes (KEGG, Kyoto, Japan) pathway annotation were summarized. The information of the draft genome sequence and functional genes for this aim were given in the Sequence Read Archive (SRA) database (National Center for Biotechnology Information, NCBI, Bethesda, MD, USA) with the accession numbers PRJNA388252 (BioProject number), SAMN07173612 (BioSample number), and SRP108180 (SRA Study number), and the supplementary data of our previous study [3]. A comprehensive analysis was conducted by incorporating the key metabolic intermediates and associated enzymes responsible for carbon sources transformation and BC biosynthesis in different KEGG pathways. The schematic diagram of carbon metabolisms and BC biosynthesis pathways in *Komagataeibacter* sp. W1 was plotted by using Microsoft PowerPoint 2016.

### 2.5. Statistical Analysis

All experiments were conducted in triplicate. The data are presented as the mean value of the triplicate with standard error. Significant differences were determined according to two-way analysis of variance (ANOVA) by Tukey’s multiple comparisons test at *p* ≤ 0.05 using GraphPad Prism (Release 6.0, La Jolla, CA, USA).

## 3. Results and Discussions

### 3.1. BC Production from Various Carbon Sources

BC production in micro-organisms is an interesting trait as this process consumes lots of energy to produce biomaterials rather than resulting in cell multiplication [31]. The possible purposes of BC production are to acquire oxygen, prevent ultraviolet damage, enhance antibiotic resistance, hold moisture, and maintain host-bacteria interactions [32,33,34]. Because glucose is the precursor for cellulose synthesis, all compounds that can be transformed to glucose are capable of BC production. Selection of the carbon substrates is one of the main requirements for efficient BC production [34]. Based on our previous study [3], the interest here in this study was to evaluate the potential of strain W1 in utilization of different carbon sources and subsequent BC production.

**Figure 1:**
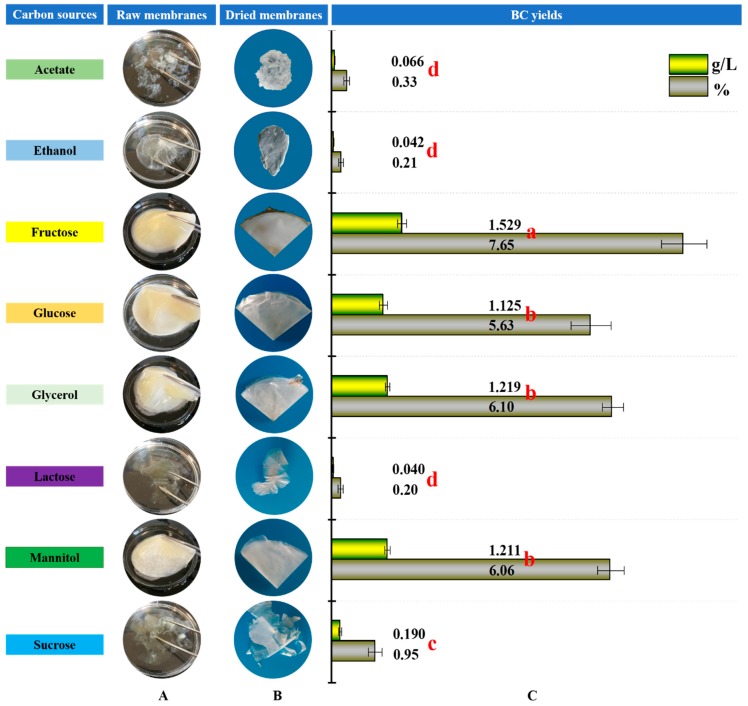
Bacterial cellulose (BC) production by *Komagataeibacter* sp. W1 grown in media spiked with different carbon sources. (**A**) the raw samples, (**B**) the samples after pre-treatment with 0.1 M NaOH for 2 h and freeze-dried for 24 h and (**C**) the BC yields. Different letters in red indicate no significant difference between the setups according to Limited Slip Differential (LSD) test (*p* ≤ 0.05).

After 14 days of incubation at 30 °C, the BCs on the surface of media containing acetate, ethanol, fructose, glucose, glycerol, lactose, mannitol and sucrose were collected and pre-treated as previously described (see details in Section 2.2). As shown in Figure 1, all eight carbon sources used in this study produced BC; among which, fructose, glucose, glycerol and mannitol produced thick membranes, but the others produced irregular and thin membranes (Figure 1A). After freeze-drying, the membranes produced from fructose, glucose, glycerol, and mannitol had a smooth surface and good mechanical properties of typical BC, as did that of sucrose although the membrane content in this group was much lower than that in the former ones (Figure 1B). However, the dried BC membranes produced from acetate, ethanol and lactose were fragile and tough (Figure 1B), indicating that the composition and structure of the BCs might be different.

To compare BC productivity, the BCs were weighed and the yields were also calculated. Similar to the findings from Figure 1A,B, the higher BC weights were observed in the groups of fructose, glucose, glycerol, and mannitol, ranging from 1.125 to 1.529 g L^−1^ (Figure 1C). The values were 5.9–38 fold of those in the media containing acetate, ethanol, lactose or sucrose (≤0.190 g L^−1^; Figure 1C). Correspondingly, strain W1 transformed 0.20–7.65% of carbon sources to BC (Figure 1C), of which, the content in fructose was significantly higher than that in glucose, glycerol, and mannitol (*p* ≤ 0.05; Figure 1C). Similarly, apparent differences in other carbon sources were also observed (Figure 1C), suggesting that the ability of strain W1 to use different carbon sources varied significantly.

To make a comparable evaluation of BC productivity in strain W1, the carbon sources used for BC production and BC yields in representative bacterial strains were summarized (Table 1). Among reported carbon sources, glucose, fructose, and mannitol are best for BC production, which is in line with our findings (Table 1; Figure 1). In some cases, however, the BC-producing bacteria prefer to utilize sucrose, lactose or a mixture of xylose and xylulose, while other carbon sources have limited use for BC production (Table 1). One possible reason is that both the structural isomers of glucose (e.g., fructose) and the precursors of glucose (e.g., glycerol and mannitol) are easy to be transformed to glucose and enter the cycles of glucose phosphorylation, glucose-6-phosphate isomerization, UDP-glucose synthesis, and extension to form BC [35]. Unlike structural isomerization or small molecule splicing, enzymatic cleavage of disaccharides is also a good strategy to produce glucose (Figure S1), explaining why some bacteria have great efficiency in sucrose and lactose utilization for BC production [12,36]. Interestingly, strain W1 was the only producer having preference to utilize fructose among the genera *Acetobacter*, *Gluconacetobacter,* and *Komagataeibacter* (Table 1). In fact, this trait is often found in other genera such as *Enterobacter* [37] and *Rhodococcus* [38]. Although strain W1 did not have great capacity to utilize carbon sources for BC production (0.015–0.547% day^−1^; Table 1) as compared to other BC producers, its preference for fructose utilization could provide useful information for further investigations.

It is also worthy to note that the medium spiked with mannitol often produced higher BC than that with glucose [13,39,40], with the highest yield of 9.58% day^−1^ (Table 1). This is different from the view that glucose is considered to be the main source for BC production [14,16]. Additionally, for a certain strain, the BC yields under different cultivation conditions are greatly different. For example, *G. xylinus* PTCC 1734 has a relative yield of 0.947% day^−1^ under agitated conditions, but which rose to 2.5% day^−1^ under static conditions (Table 1), suggesting the significant effects of cultivation methods on BC production [12]. Between above two conditions, the most suitable carbon sources for BC production were sucrose and mannitol, respectively (Table 1). As regards the BC yields with different strains of *K. xylinus* (formerly *A. xylinus* or *G. xylinus*), the data vary from 0.071% to 5.29% day^−1^ (Table 1). The above mentioned observations suggest that there is no similar pattern of bacterial behavior to utilize carbon sources for BC production, selecting the most appropriate carbon source for BC production in an individual strain is very important [41].

In our study, all used carbon sources were sugars except for acetate and ethanol, which are well-known substrates responsible for enhancing ATP production and inhibiting the anti-BC production processes [17]. The studies focusing on acetate and (or) ethanol addition and their consequent effects on BC production have been well documented [17,18,19,20]. Although some studies have tried to produce BC using acetate or ethanol as a sole carbon source, the BC yields are much lower than for sugars [16,36]. This was supported by our study (Figure 1C; Table 1), implying that both acetate and ethanol mainly serve as BC-producing enhancers rather than transforming to BC directly.

### 3.2. BC Morphology and Microfibril Analysis by SEM Observation

**Figure 2:**
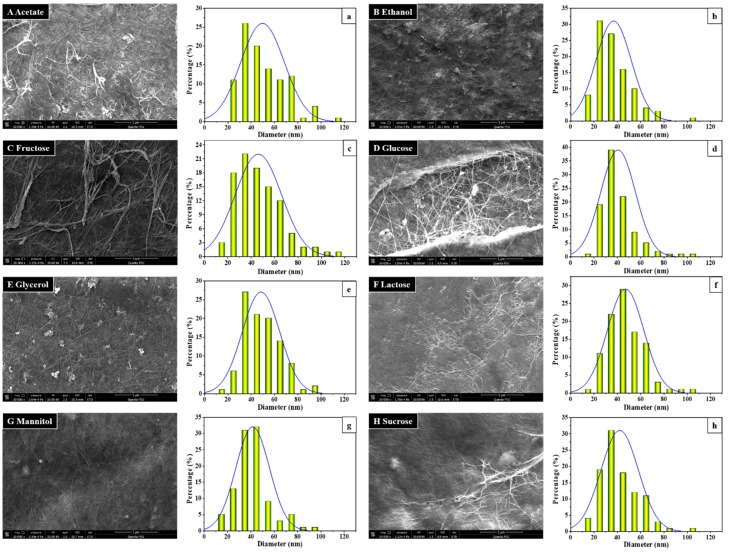
Morphology (**A**–**H**) and diameter distribution (**a**–**h**) of the BC produced by *Komagataeibacter* sp. W1 grown in the media spiked with different carbon sources. While the BC morphology was observed with scanning electron microscopy (SEM) with a spot of 3.0, high voltage of 15 KeV, and magnification of 20,000×, the diameter calculation was performed on Nano Measurer 1.2 by calculating 100 nanofibrils randomly on the SEM images.

The morphology and structural characteristics of BC membranes produced by *Komagataeibacter* sp. W1 from various carbon sources were evaluated by SEM. As can be seen in Figure 2A–H, all carbon sources used in this study produced BC pellicles with a dense morphology. Our findings were similar to *K. xylinus* strains KX, TISTR 086, 428, 975, 1011, and B-12068 [12,47] but different from *K. medellinensis* and *K. xylinus* CH001 [2,19], the latter two produced a less dense network of microfibrils with high porosity of up to 60%. This indicates that different bacteria or bacterial strains may have a different interwoven pattern to produce BC.

Due to the indistinguishable differences in BC morphology, we also calculated the BC diameter distribution by Nano Measurer 1.2 based on SEM images. The results showed that all carbon sources could produce 30 nm or smaller microfibrils, with an average diameter of 40–50 nm except for ethanol (Figure 2a–h). Our data were in line with the study from Volova et al. [47], suggesting that the small microfibrils were also likely to be associated with a lower density of BC as described previously. It was apparent that the visible component of BC in our study was cellulose ribbons (often 40–60 nm) rather than nanofibrils because the diameter of the sub-elementary fibrils is 3–4 nm [49,50]. Although the appearances of BCs produced from acetate, ethanol, lactose, and sucrose were different from other substrates (Figure 1), both SEM imaging and diameter distribution analysis verified that all carbon sources used in this study produced typical BC (Figure 2). However, the reasons influencing BC production efficiency in *Komagataeibacter* sp. W1 still warrant future investigations.

### 3.3. Crystalline Differences of BC Membranes Produced from Various Carbon Sources

**Figure 3:**
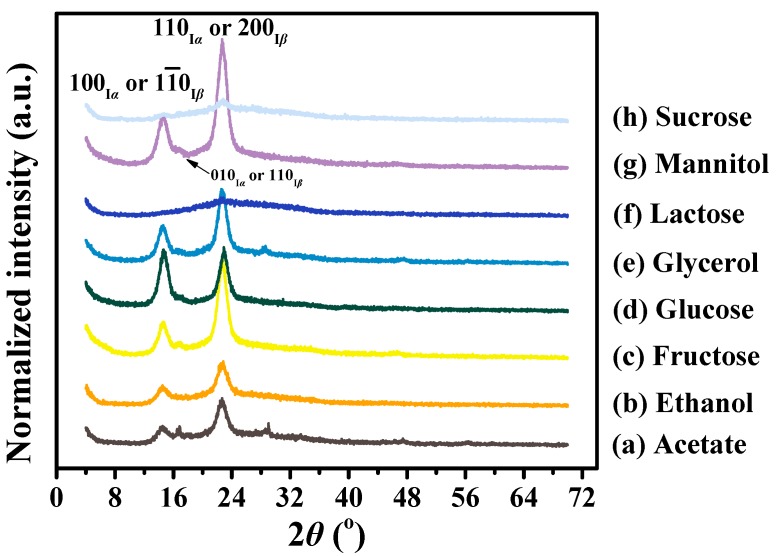
Comparative X-ray diffraction (XRD) analysis of the BC produced by *Komagataeibacter* sp. W1 grown in the media spiked with various carbon sources. The XRD pattern was obtained using nickel filtered copper *K*_α_ radiation, with 0.1° steps, from 4° to 70° (2*θ*).

It is well-known that BC is a homogeneous polycrystalline macromolecular compound composed of ordered crystalline and less ordered amorphous regions [51]. To determine the crystal structure and the crystalline contents of BC, all samples were analyzed by XRD. Figure 3 shows that different carbon sources result in different diffraction profiles. Specifically, the BCs produced from fructose, glucose, glycerol, and mannitol displayed two typical peaks at 2*θ* of 14.5° and 22.7° with strong intensity and a weak peak at 2*θ* of 16.6° (Figure 3c–e,g). While the two broad peaks were assigned to (100) and (110) planes of cellulose *I_α_* or (1$\bar{1}$0) and (200) planes of cellulose *I_β_*, respectively, the weak peak indicated the presence of the (010) plane of cellulose *I_α_* or the (110) plane of cellulose *I_β_* (Figure 3) [52]. The higher intensity of the peak 14.5° than that of the peak 16.6° made it clear that the content of *I_α_* was higher than *I_β_* [53] and the shape of the cellulose crystallites was rectangular rather than square cross-sectional [51], in agreement with the cases of Singhsa et al. [12] and Keshk and Sameshima [54]. It was interesting to note that although acetate and ethanol produced limited BCs, they showed a similar XRD pattern to the BCs produced from the above-mentioned sugars (Figure 3a,b). However, only one weak peak was observed on BC membranes produced from lactose and sucrose (Figure 3f,h). At around 2*θ* of 28.6°, a weak peak was found in the acetate and glycerol groups, which has been scarcely reported and needs further investigations (Figure 3a,e). Our data indicated that all carbon sources could produce BC with high crystallinity except for lactose and sucrose.

As shown in Table 2, the interplanar crystal distance (i.e., *d*-spacing) of each peak of most of the BCs was the same, assuming that peak shifts at 2*θ* of 14.5^o^ did not occur and different BCs had the same contents of cellulose *I_α_* [23]. Due to the absence of the (100_*Iα*_) or (1$\bar{1}$0_*Iβ*_) peak in the lactose group and the (010_*Iα*_) or (110_*Iβ*_) peak in ethanol, lactose and sucrose groups, all the corresponding *d*-spacing and ACSs were not available (Table 2). Unlike *d*-spacing, however, the ACS varied significantly in different groups (Table 2). For instance, the ACSs of peak 1 in fructose, glucose, glycerol, and mannitol were similar, at 7.9–8.8 nm, which reduced to 6.7–6.9 nm in acetate and ethanol and increased to 23.6 nm in sucrose (Table 2). The lower or higher ACSs in acetate, ethanol, lactose, and sucrose might be attributed to the irregular textile structures of BC microfibrils (Figure 1 and Figure 2). Similar to peak 1, most BCs had a typical ACS in peak 3, but the ACSs in lactose and sucrose were much lower than that in the others (Table 2). However, since peak 2 was weak, the corresponding ACSs did not follow similar trends as for peak 1 and peak 3 (Figure 3; Table 2), which again verified the high contents of *I_α_* in all groups as previously described.

Besides *d*-spacing and ACS, we also determined C.I. and% crystalline of BC samples produced from different carbon sources. In general, the C.I. ranged from 0.64–0.89, the highest and the lowest being in mannitol and lactose, respectively (Table 2). Correspondingly, all BCs had a high crystallinity ranging from 83–90% except for 73% for lactose and 74% for sucrose, respectively (Table 2). This followed similar trends as for *d*-spacing and ACS (Table 2) but was different from BC yields (Figure 1). Moreover, our data showed a similar crystallinity to that reported by previous studies [2,14,23]. In most cases, a lower ACS corresponded to a higher BC crystallinity (Table 2), which was supported by a recent study from Meza-Contreras et al. [55]. Because the peak corresponding to the (200) lattice plane often shows the highest intensity in the diffraction patterns of native BC (Figure 3) and plays an important role in higher crystallinity and smaller crystallite size [19], mannitol could be an ideal carbon source for BC production in *Komagataeibacter* sp. W1.

### 3.4. Functional Groups and Cellulose Types Characterization Based on FTIR Analysis

**Figure 4:**
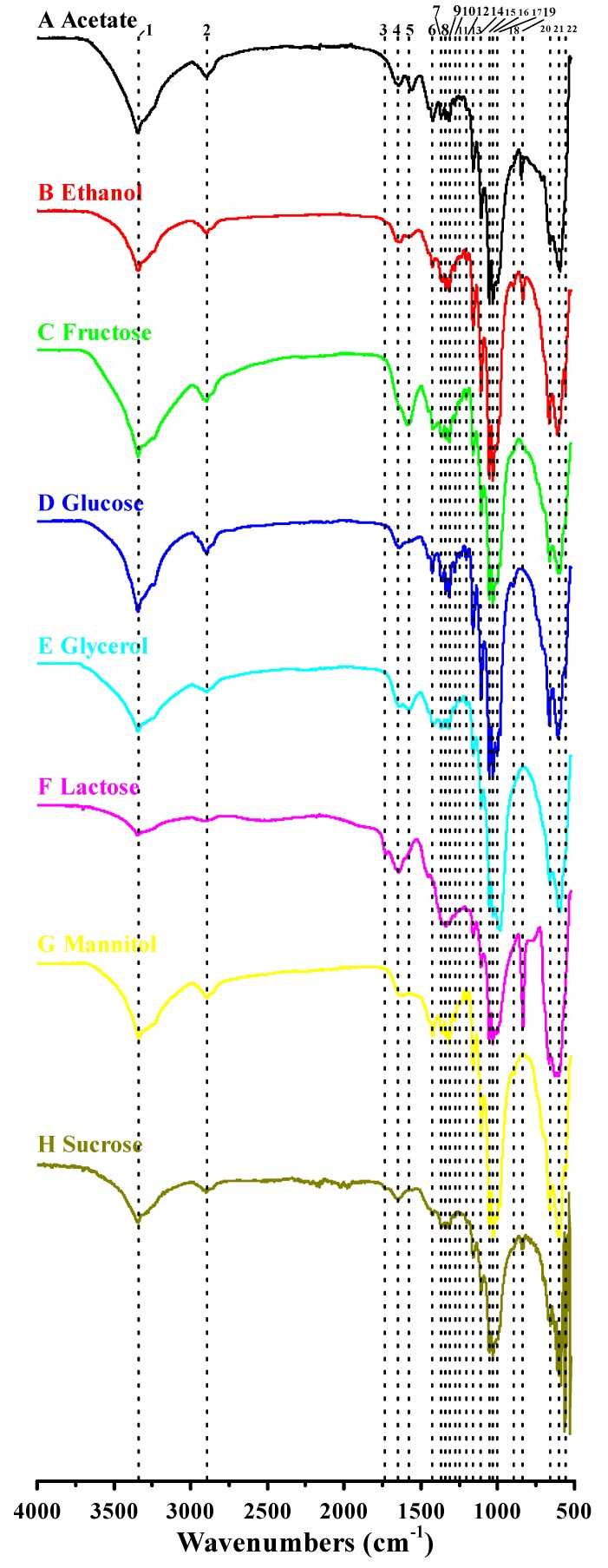
Comparative Fourier transform infrared (FTIR) analysis of the BC produced by *Komagataeibacter* sp. W1 grown in media spiked with various carbon sources. The analysis was conducted on a Nicolet iS5 in the Attenuated Total Reflectance (ATR) mode with 32 scans per measurement between 400 and 4000 cm^−1^. The detailed information of peaks 1–22 is given in Table 3.

The FTIR spectra of BCs prepared from different carbon sources are shown in Figure 4. Although different carbon sources resulted in great differences in BC yields (Figure 1C), and XRD patterns of BCs also varied significantly (Figure 3), all FTIR spectra exhibited several typical vibration bands with little difference (Figure 4), implying the same chemical structure for the different BCs prepared from various carbon sources [2]. Of the 22 peaks, most have been assigned to certain functional groups in previous studies. For example, several typical adsorptions associated with O–H stretching at around 3345 cm^−1^, C–H stretching at around 2900 cm^−1^, C–O–H antisymmetric bridge stretching of 1,4-β-glucoside at around 1160 cm^−1^ and antisymmetric out-of-phase ring stretching of β-glucosidic linkages between glucose units at around 900 cm^−1^ were observed (Figure 4; Table 3). Similarly, we also found other adsorptions due to O–H bending at around 1360, 1280, and 1205 cm^−1^, O–H in-plane bending at around 1430 and 1335 cm^−1^, C–O bending at around 1108, 1055, and 1031 cm^−1^, and O–H out-of-phase bending at the wavenumbers below 660 cm^−1^ (Figure 4; Table 3). However, the adsorptions at 1430, 1335, 1108, 1055, and 1031 cm^−1^ might also be assigned to CH_2_ symmetric bending, C–H deformation, C–C bonds of the monomer units of polysaccharide, and C–O–C pyranose ring skeletal vibration, respectively (Table 3). Our study revealed that the BCs produced by *Komagataeibacter* sp. W1 were mostly composed of cellulose I (adsorptions at around 3345, 1430, 1160, and 900 cm^−1^) with few cellulose II (adsorption at around 1335, 1315, and 1280 cm^−1^ and a blue-shift of wavenumber from 1430 to around 1425 cm^−1^) (Table 3) [3].

As noted previously, BC characterization is often performed by using XRD and ^13^C-NMR methods [46,55,56]. However, due to the overlap of cellulose *I_α_* and *I_β_* reflections, it is difficult to differentiate the two allomorphs by only determining the XRD peak positions [12]. Molina-Ramírez et al. [2] showed that the FTIR could help distinguish *I_α_* allomorph (around 3240 and 750 cm^−1^) from *I_β_* allomorph (around 3270 and 710 cm^−1^). The *I_α_* fractions of BCs produced from fructose, glucose, and sucrose in *K. medellinensis* were from 0.70 to 0.74 [2], which were higher than *A. xylinus* 23769 of 0.36–0.43 [23], *A. xylinus* strains ATCC 10245, IFO 13693, IFO13772, IFO13773, IFO14815, and IFO15237 of 0.38–0.43 [54], and *Komagataeibacter* sp. W1 of 0.51 for all groups in our study. However, our data were in accordance with the well-known fact that for *I_α_* it is as high as about 64% in BC [57]. We hypothesized that strain W1 possessed the capacity to produce both allomorphs at the same time, and the ability to produce *I_α_* and *I_β_* was stable irrespective of the spiked carbon sources.

### 3.5. Insights into the Biochemical Pathways for Carbon Sources Utilization and BC Synthesis

In micro-organisms, several metabolic pools are involved in BC biosynthesis. One direct pathway to produce BC is to pre-generate hexose phosphate by the phosphorylation of exogenous hexoses [35]. To this aim, the isomers fructose and glucose are ideal carbon sources for BC production, which was supported by our findings (Figure 1 and Figure S1). As to other carbon sources, transforming to the above two hexoses is the first and important step to produce BC through the pentose cycle and gluconeogenic pathway [58]. In addition to physio-biochemical observation and evaluation of BC synthesis in micro-organisms, it is also important to understand the metabolic network of carbon sources [41]. However, this is scarcely reported in existing studies.

**Figure 5:**
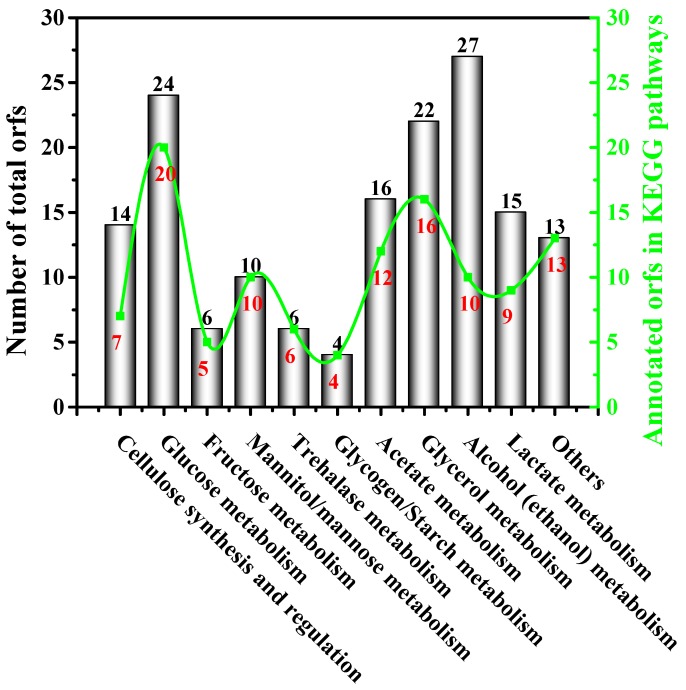
The number of orfs associated with carbon source metabolisms and BC biosynthesis and regulation. The orfs shown here were retrieved from the genes and corresponding protein annotation data. While the numbers on the top of the bars indicate the total number of predicted genes involved in carbon source metabolisms and BC biosynthesis and regulation, the ones below the line indicate the number of the genes that can be annotated to certain Kyoto Encyclopedia of Genes and Genomes (KEGG) pathways. It’s worthy to note that the description ‘others’ indicates the key metabolic intermediates during the transformation between glucose and glycerol or fructose and glycerol. More details can be found in Tables S1 and S2.

Our previous study obtained the genome information of strain W1 and annotated some associated enzymes in glucose transformation and BC synthesis [3]. To explore how strain W1 utilized other carbon sources and then produced BC, this study also summarized the orfs responsible for various carbon source metabolisms and BC production. As shown in Figure 5 and Table S1, strain W1 had 157 orfs corresponding to various carbon source metabolisms, most of which had been studied in this work except for mannose, glycogen/starch, and trehalose. In fact, besides those orfs responsible for encoding the enzymes involved in direct glucose transformation and cellulose synthesis and regulation, we also found more indirect orfs aiming for these processes (Table S1). In general, strain W1 had 27 orfs associated with alcohol (ethanol) metabolism, being the highest among 11 groups, followed by glucose metabolism of 24 and glycerol metabolism of 22 (Figure 5). This was understandable because strain W1 was an acetic bacterium used for vinegar production (alcohol metabolism) and a typical BC-producer capable of BC production (glucose metabolism) [3]. Unlike normal carbon substrates, glycerol not only can be transformed to glucose, it is also easily degraded and used as a source of energy to enhance the TCA cycle [23,24,41]. It was interesting to note that both orfs corresponding to fructose and mannitol metabolism were less than for the others except for trehalose and glycogen/starch (Figure 5). Apparently, the number of orfs did not follow the trend of BC productivities as described previously (Figure 1C), suggesting that carbon source metabolism might also be impacted by other factors such as enzymatic activity, substrate preference, and metabolic fluxes [2,59].

**Figure 6:**
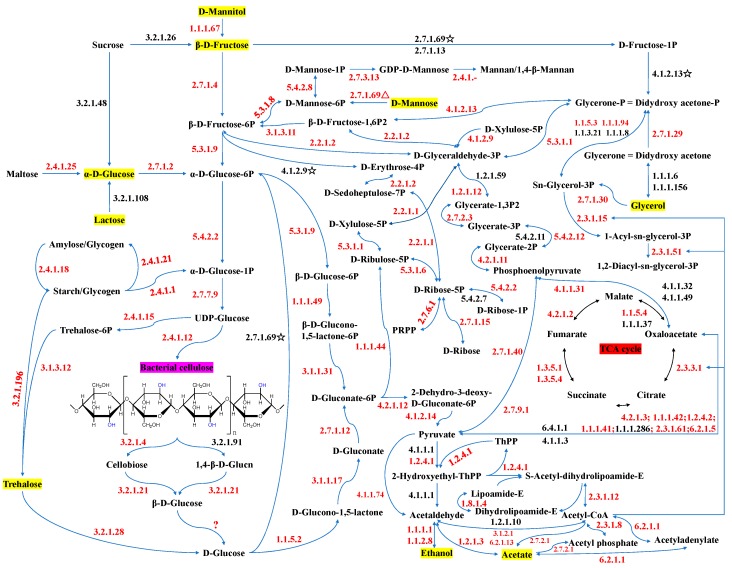
Schematic diagram of carbon metabolism and BC biosynthesis pathways in *Komagataeibacter* sp. W1. The comprehensive analysis was conducted by incorporating the key metabolic intermediates and associated enzymes responsible for carbon source transformation and BC biosynthesis in different KEGG pathways. The numbers in the figure are the Enzyme Commission number, while the red and black ones indicate the associated enzymes present and absent respectively in *Komagataeibacter* sp. W1. The asterisks indicate the enzymes absent in the labeled pathways but present in other pathways in our study, thus it is unknown whether they work in the labeled pathways. The question mark indicating whether the pathway is present, is unclear based on our data and remains for future investigations. More information of the enzymes and the associated genes and pathways (ko numbers) are listed in Table S2. P, phosphate; GDP, guanosine diphosphate; UDP, uridine diphosphate; PRPP, phosphoribosyl pyrophosphate; ThPP, thiamine diphosphate; TCA, tricarboxylic acid cycle; Acetyl-CoA, acetyl coenzyme A.

To have a full understanding of carbon source metabolism and BC production in strain W1, we further provided an overview of the orfs that could be annotated to certain KEGG pathways (Figure 5; Table S2). Based on the coupling analysis of the orfs-KEGG pathway, the schematic diagram of carbon source metabolism and BC biosynthesis pathways in *Komagataeibacter* sp. W1 were also provided (Figure 6). As expected, most orfs could be annotated to a certain KEGG pathway (Figure 5), and all corresponding enzymes are given in Table S2 and labeled in Figure 6. Specifically, fructose, mannitol, and glycerol could be enzymatically transformed to glucose, and then produce BC, through the pentose phosphate pathway or gluconeogenesis pathway [59], but lactose and sucrose did not (Figure 6). Although acetate and ethanol were able to generate acetyl coenzyme A (acetyl-CoA) and functioned in the TCA cycle and glycerol transformation, we did not find any possible pathway linking them to glucose or other sugars (Figure 6). Since media glucose was removed before seed solution transfer, it was possible that both acetate and ethanol were not the precursors of glucose and the few BCs observed in our study might be due to the release of cell glucose and subsequent BC production (Figure 1). We also noticed that glycerol, mannitol, and fructose had two pathways (i.e., the pyruvate pathway and glycerate-phosphate pathway) to produce oxaloacetate and enter the TCA cycle [24], thereby enhancing energy generation and carbon sources utilization for BC production (Figure 1 and Figure 6). However, our results were different from Molina-Ramírez et al. [2] in that glucose obtained 86% higher BC than fructose, again showing that different bacterial strains had different traits for carbon source utilization and BC production (Table 1).

## 4. Conclusions

In this study, *Komagataeibacter* sp. W1, which is a typical BC-producer, was subjected to media spiked with various carbon sources including acetate, ethanol, fructose, glucose, lactose, mannitol, and sucrose and the BC productivity, BC characteristics and biochemical transformation pathways associated with carbon source transformation and BC production were investigated. This strain preferred to use fructose, glucose, glycerol, and mannitol for BC production, with the highest BC yield being 1.529 g L^−1^ on fructose. SEM analysis suggested that the membranes produced from all carbon sources were composed of nanofibrils with an average diameter of 35–50 nm, which is a typical characteristic of BC, consistent with the results from XRD and FTIR analyses. Based on genome annotation and KEGG analysis, all biochemical transformation pathways associated with the utilization of and BC production from fructose, glucose, glycerol, and mannitol were found. Our data provided suggestions to further investigations of strain W1 to produce BC by using the above carbon sources and gave clues on understanding how this strain produces BC at the metabolic pathway scale.

## Figures and Tables

**Table 1:** The carbon sources used for bacterial cellulose (BC) production and BC productivity in representative bacterial strains.

Bacteria Names	Cultivation Conditions	Carbon Sources	Concentration (%, *w/v*)	Relative Yields (% Day^−1^) ^a^	References
*Acetobacter* sp. S-35	Static, 28 °C, 3 days	Sucrose, lactate, glucose, mannitol ^b^, gluconate, glycerol, dulcitol, maltose, lactose, sorbose, ribose, arabinose, xylose, fructose, galactose	4% except for dulcitol and sorbose with 2%	up to 9.58	Kojima et al. [40]
*Acetobacter hansenii* ATCC 10821	Static, 30 °C, 30 days	d-glucose ^b^, d-xylose, d-xylulose, d-xylose/d-xylulose	2%	0.037–1.087	Ishihara et al. [42]
*Acetobacter lovaniensis* HBB5	Static, 30 °C, 7 days	Glucose ^b^, sucrose, fructose, ethanol	2%	0.008–0.029	Çoban and Biyik [16]
*Acetobacter pasteurianus* IFO 14814	Static, 30 °C, 30 days	d-glucose ^b^, d-xylose, d-xylulose, d-xylose/d-xylulose	2%	0.130–0.167	Ishihara et al. [42]
*Acetobacter xylinum* ATCC 10245	Static, 30 °C, 14 days	Glucose	1.5%	5.29	Hassan et al. [39]
*Acetobacter xylinum* ^c^	NM d, 35 °C, 14 days	Sucrose ^b^, glucose, mannitol ^b^, sorbitol, galactose, lactose, acetic acid, maltose	5–7%	≥0.057 ^e^	Ramana et al. [36]
*Acetobacter xylinus* IFO 15606	Static, 30 °C, 30 days	d-glucose, d-xylose, d-xylulose, D-xylose/d-xylulose ^b^	2%	0.147–0.347	Ishihara et al. [42]
*Chromobacterium violaceum* ATCC 12472	Static, 32 °C, 3 days	Glucose	2%	NM	Recouvreux et al. [43]
*Enterobacter amnigenus* GH-1	Static, 30 °C, 14 days	d-Glucose, d-fructose ^b^, lactose, mannitol, inositol, sucrose, maltose, glycerol	2%	1.0	Hungund and Gupta [44]
*Enterobacter* sp.	Agitated, 30 °C, 24 days	Glucose	2%	0.5	Ago et al. [37]
*Gluconacetobacter intermedius* NEDO-01	Static, 30 °C, 3 days Agitated, 30 °C, 4 days	Glycerol	2%	NM 4.25	Kose et al. [15]
*Gluconacetobacter sacchari*	Static, 30 °C, 4 days	Glucose	2%	3.375	Trovatti et al. [45]
*Gluconacetobacter xylinus* ATCC 53524	Static, 30 °C, 2 or 4 days	Mannitol ^b^, glucose, glycerol, fructose, sucrose ^b^, galactose	2%	5.10 or 4.79	Mikkelsen et al. [13]
*Gluconacetobacter xylinus* CH001	Static, 28 °C, 14 days	Xylose	1–3%	up to 0.482	Yang et al. [19]
*Gluconacetobacter xylinus* PTCC 1734	Agitated, 28 °C, 7 days	Mannitol, sucrose ^b^, glucose	2–5%	~0.947	Mohammadkazemi et al. [28]
*Gluconacetobacter xylinus* PTCC 1734	Static, 28 °C, 20 days	Glucose, fructose, mannitol ^b^, sucrose, glycerol	2%	up to 2.5	Tabaii and Emtiazi [41]
*Komagataeibacter medellinensis*	Static, 28 °C, 8 days	Fructose, glucose ^b^, sucrose	2%	0.238–1.75	Molina-Ramírez et al. [2]
*Komagataeibacter rhaeticus* PG2	Static, 28 °C, 15 days	Fructose, lactose, xylose, sucrose, galactose, mannitol, sorbitol, and glycerol	2%	up to 2.3	Thorat and Dastager [46]
*Komagataeibacter saccharivorans* PE 5	Static, 30 °C, 14 days	Mannitol	1.5%	6.00	Hassan et al. [39]
*Komagataeibacter xylinus* KX, TISTR 086, 428, 975 and 1011	Static, 30 °C, 7 days Agitated, 30 °C, 7 days	Glucose ^b^, fructose, lactose ^b^, maltitol, sucralose, xylitol	5%	0.326–0.526 up to 1.34	Singhsa et al. [12]
*Komagataeibacter xylinus* B-12068	Static, 30 °C, 7 days	Glucose ^b^, sucrose, galactose, maltose, mannitol	2%	0.071–1.571	Volova et al. [47]
*Rhodococcus* sp. MI 2	Static, 25 °C, 14 days	Glucose, fructose ^b^, sucrose, lactose, sorbitol, and mannitol	2%	~2.25	Tanskul et al. [38]
*Saccharomyces cerevisiae* CGMCC1670	Static, 30 °C, 22 days	Glucose	5%	~0.118	Tan et al. [48]
*Komagataeibacter* sp. W1	Static, 30 °C, 14 days	Acetate, ethanol, fructose, glucose, glycerol, lactose, mannitol ^b^, sucrose	2% ^f^	0.015–0.547	This study

^a^ Since the amount of carbon sources used for BC production was different, the BC productivity was calculated based on the initial and final contents of carbon sources and BC within the incubation time. ^b^ Carbon source for the highest BC production. ^c^ The bacteria have been re-classified into the genus *Komagataeibacter*. ^d^ Not mentioned. ^e^ Both sucrose and mannitol (60–70 g L^−1^) produced similar amounts of BC with the addition of peptone or casein hydrolysate. ^f^ All carbon sources were normalized to glucose based on C element amount.

**Table 2:** *D*-spacing, apparent crystal size (ACS), crystallinity index (C.I.). and % crystalline of BC samples produced from different carbon sources.

Carbon Sources	Peak 1 (100_*Iα*_ or 1$\bar{1}$0_*Iβ*_)		Peak 2 (010_*Iα*_ or 110_*Iβ*_)		Peak 3 (110_*Iα*_ or 200_*Iβ*_)		At 2*θ* Scale		C.I.	% Crystalline
	*d*-Spacing (nm)	ACS (nm)	*d*-Spacing (nm)	ACS (nm)	*d*-Spacing (nm)	ACS (nm)	*I_am_*	*I_ma_*		
Acetate	0.60	6.7	0.53	15.5	0.39	7.8	133	636	0.79	83
Ethanol	0.60	6.9	– ^a^	–	0.39	7.2	126	602	0.79	83
Fructose	0.60	8.2	0.53	9.4	0.39	8.9	165	1323	0.88	89
Glucose	0.60	8.8	0.53	6.8	0.39	9.2	132	844	0.84	86
Glycerol	0.60	8.6	0.53	10.7	0.39	8.9	140	978	0.86	87
Lactose	–	–	–	–	0.39	1.8	99	273	0.64	73
Mannitol	0.60	7.9	0.53	8.0	0.39	8.8	185	1674	0.89	90
Sucrose	0.60	23.6	–	–	0.39	4.6	112	322	0.65	74

^a^ Since the peak was weak, both the *d*-spacing and ACS were not available.

**Table 3:** Fourier transform infrared (FTIR) analysis of functional groups on BC produced from different carbon sources.

Peak Number	Wavenumber (cm^−1^)								Functional Groups
	Acetate	Ethanol	Fructose	Glucose	Glycerol	Lactose	Mannitol	Sucrose	
1	3344	3343	3343	3343	3344	3345	3341	3346	O–H stretching vibration
2	2895	2896	2895	2895	2895	2897	2894	2899	C–H stretching of CH_2_ and CH_3_ groups
3	– ^a^	–	–	–	–	1730	–	1734	UK ^c^
4	1648	1645	–	1645	1645	1649	1642	1647	H–O–H bending of absorbed water
5	1564	1573	1574	–	1574	–	–	–	UK
6	1424	1427	1423	1427	1423	–	1426	1423	CH_2_ symmetric bending or O–H in plane bending
7	1361	1360	1361	1360	1361	1355	1361	1361	C–H bending
8	1336	1335	1335	1335	1336	1336	1335	1335	C–H deformation or O–H in-plane bending
9	1315	1315	1315	1315	1315	1315	1315	1315	Out-of-plane wagging of the CH_2_ groups
10	1280	1281	1280	1280	1281	1281 ^b^	1280	1280 ^b^	C–H bending
11	1248	–	1249	1249	1249 ^b^	–	–	1249 ^b^	UK
12	1203 ^b^	1205	1205	1205	1204	1203 ^b^	1205	1202 ^b^	C–H bending
13	1160	1161	1161	1161	1161	1160	1160	1161	C–O–C antisymmetric bridge stretching of 1, 4-β-d-glucoside
14	1108	1108	1108	1108	1108	1109	1108	1108	C–C bonds of the monomer units of polysaccharide or C–O bending vibration
15	1055	1055	1054	1054	1055	1056	1055	1054	The bending of C–O–H bond of carbohydrates or C–O–C pyranose ring skeletal vibration
16	1031	1031	1031	1031	1031	1032	1030	1032	
17	1003 ^b^	1005 ^b^	1003 ^b^	1004	997	1002 ^b^	1003	1002 ^b^	UK
18	899 ^b^	899	–	899	–	–	895	–	Antisymmetric out-of-phase ring stretching of β-glucosidic linkages between the glucose units
19	847	835	–	–	–	836	–	836	UK
20	660	664	663	664	662	666	663	657	O–H out-of-phase bending vibration
21	600	609	602	609	597	602	601	597	
22	563	559	557	558	563	564	558	562	

^a^ Not detected. ^b^ The peaks were weak. ^c^ Unknown.

## Supplementary Materials

The following are available online at http://www.mdpi.com/2073-4360/10/9/963/s1, Figure S1: Structures of various carbon sources (A–H) and bacterial cellulose (I), Table S1: Open reading frames and corresponding proteins information based on NCBI non-redundant protein (Nr) and Swissprot annotations, Table S2: Open reading frames and corresponding proteins information based on KEGG pathway annotation.
