# Supplementary material for: Insights into Bacterial Cellulose Biosynthesis from Different Carbon Sources and the Associated Biochemical Transformation Pathways in Komagataeibacter sp. W1
Source: Polymers (Basel). 2018 Aug 31;10(9):963. doi: 10.3390/polym10090963 (PMC6403882; doi:10.3390/polym10090963)
Supplement: Supplementary file 1 [file polymers-10-00963-s001.pdf]

Supplementary Materials: Insights into Bacterial Cellulose Biosynthesis from Different Carbon Sources and the Associated Biochemical Transformation Pathways in *Komagataeibacter* sp. W1

Shan-Shan Wang, Yong-He Han, Jia-Lian Chen, Da-Chun Zhang, Xiao-Xia Shi, Yu-Xuan Ye, Deng-Long Chen and Min Li

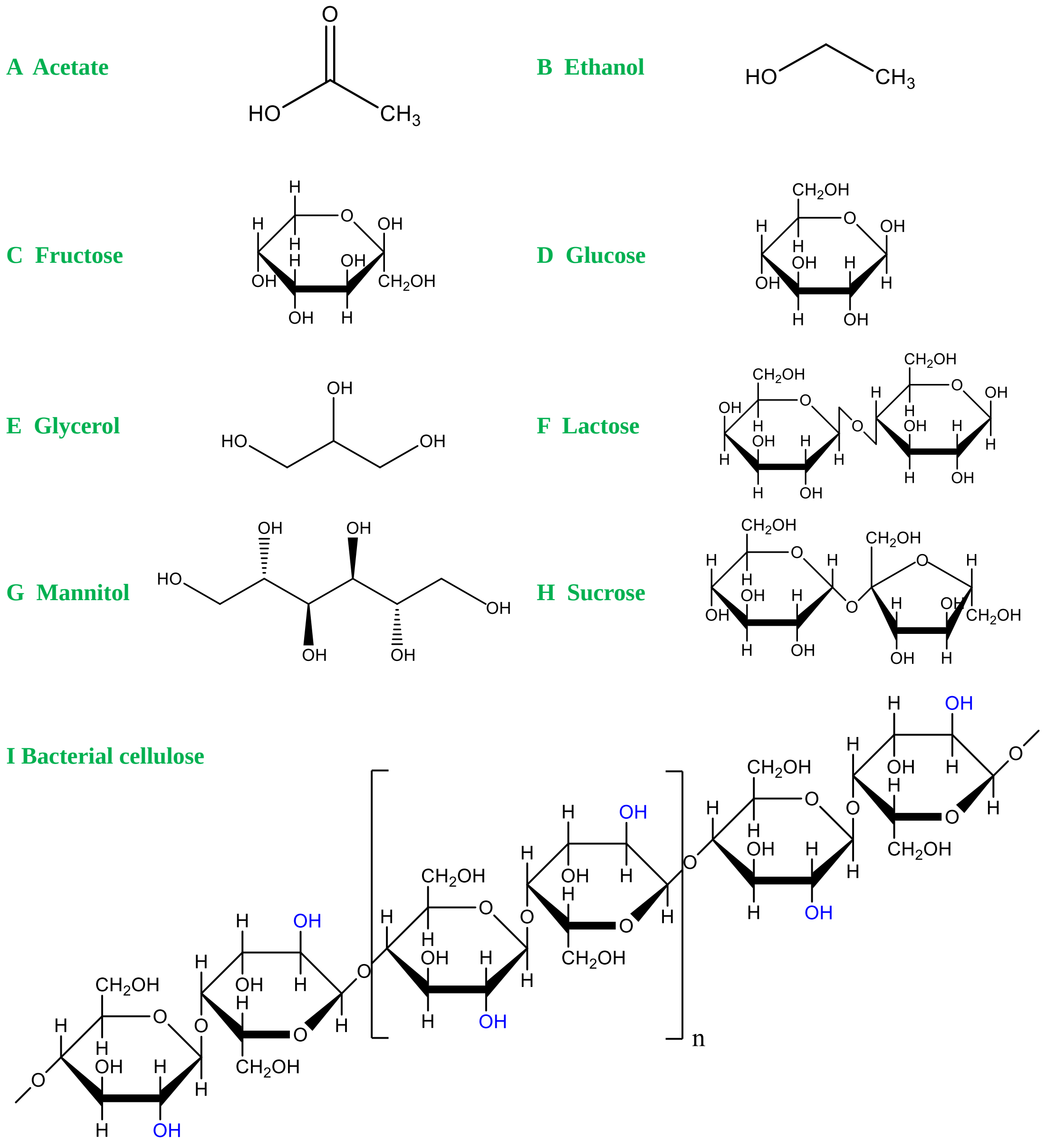

Figure S1. Structures of various carbon sources (A–H) and bacterial cellulose (I).

Table S1. Open reading frames and corresponding proteins information based on NCBI non-redundant protein (Nr) and Swissprot annotations.

Open reading frames number	Amino acid length (aa)	NR top hit	Similarity (%)	Swissprot top hit	Similarity (%)	Protein names
**A Cellulose metabolism (7/14)[a]**						
**Synthesis (4/10)**						
orf0140[b]	1530	–[f]	–	Q9WX75.1	79	Cellulose synthase catalytic subunit [UDP-forming]
orf0492[c]	1328	WP_026019532.1	100	Q9WX71.1	88	Cellulose synthase [*Komagataeibacter europaeus*]; Cellulose synthase 2 operon protein C/Flags: Precursor [*Komagataeibacter xylinus*]
orf0493[c]	386	WP_019084639.1	100	Q9WX70.1	94	Acyltransferase [*Komagataeibacter europaeus*]; Putative membrane-bound transacylase BcsY [*Komagataeibacter xylinus*]
orf0494[c]	223	WP_019090487.1	100	Q9WX69.1	99	BcsX [*Komagataeibacter europaeus*]; Protein BcsX [*Komagataeibacter xylinus*]
orf0495[bc]	1558	–	–	Q9RBJ2.1	92	Putative cellulose synthase 2=Cellulose synthase catalytic subunit [UDP-forming]
orf1576	321	WP_019085254.1	92	Q76KK0.1	60	Hypothetical protein [*Komagataeibacter europaeus*]; Cellulose-complementing protein [*Komagataeibacter xylinus*]
orf1578[bd]	691	WP_026018529.1	100	Q9WX61.1	96	Cellulose synthase [*Komagataeibacter europaeus*]; Cellulose synthase 1 catalytic subunit [*Komagataeibacter xylinus*]
orf1579[bd]	802	–	–	Q9WX62.1	97	Cyclic di-GMP-binding protein=CDGBP=Cellulose synthase regulatory subunit=Cellulose synthase protein B/Flags: Precursor
orf1580[d]	1293	WP_026019647.1	100	Q9WX63.1	96	Cellulose synthase 1 operon protein C/Flags: Precursor [*Komagataeibacter xylinus*]; Cellulose synthase [*Komagataeibacter europaeus*]
orf1581[d]	156	WP_010507046.1	100	Q9WX64.1	100	Cellulose synthase operon protein D [*Komagataeibacter xylinus*]; cellulose synthase [*Komagataeibacter europaeus*]
**Regulation (3/4)**						

orf1575[b]	354	WP_019085253.1	100	P37696.1	86	Endoglucanase [*Komagataeibacter europaeus*]; Probable endoglucanase=Cellulase=Endo-1,4-beta-glucanase/Flags: Precursor [*Komagataeibacter hansenii*]
orf1582[b]	734	WP_010507044.1	100	Q5BFG8.1	53	Beta-glucosidase [*Komagataeibacter europaeus*]; Beta-glucosidase B=Beta-D-glucoside glucohydrolase B=Cellobiase B=Gentiobiase B
orf1669[b]	325	WP_019086736.1	100	P58599.1	60	Endoglucanase [*Komagataeibacter europaeus*]; Endoglucanase= Cellulase=Endo-1,4-beta-glucanase/Flags: Precursor [*Ralstonia solanacearum* GMI1000]
orf2722	698	WP_010508536.1	100	Q9WX70.1	51	Acyltransferase [*Komagataeibacter europaeus*]; Putative membrane-bound transacylase BcsY [*Komagataeibacter xylinus*]
**B Glucose metabolism (20/24)**						
orf0040[b]	493	WP_010509282.1	100	Q9Z3S2.2	69	Glucose-6-phosphate 1-dehydrogenase [*Komagataeibacter europaeus*]; Glucose-6-phosphate 1-dehydrogenase=G6PD [*Sinorhizobium meliloti* 1021]
orf0238[b]	191	WP_019084469.1	100	P39208.1	64	Carbohydrate kinase [*Komagataeibacter europaeus*]; Thermosensitive gluconokinase=Gluconate kinase 1 [*Escherichia coli* K-12]
orf0431	415	WP_019090457.1	100	–	–	Gluconolactonase [*Komagataeibacter europaeus*]
orf0534[b]	126	WP_026018750.1	100	P39208.1	59	Carbohydrate kinase [*Komagataeibacter europaeus*]; Thermosensitive gluconokinase=Gluconate kinase 1 [*Escherichia coli* K-12]
orf1086[b]	555	WP_019091484.1	100	P38569.1	100	Phosphoglucomutase [*Komagataeibacter europaeus*]; Phosphoglucomutase=PGM=Glucose phosphomutase [*Komagataeibacter xylinus*]
orf1122[b]	280	WP_019084882.1	100	P27897.1	97	UTP-glucose-1-phosphate uridylyltransferase [*Komagataeibacter europaeus*]; UTP-glucose-1-phosphate uridylyltransferase=Alpha-D-glucosyl-1-phosphate uridylyltransferase=UDP-glucose pyrophosphorylas
orf1159[b]	770	WP_010507847.1	100	P15877.3	78	Glucose dehydrogenase [*Komagataeibacter europaeus*]; Quinoprotein glucose dehydrogenase=Glucose dehydrogenase [*Escherichia coli* K-

						12]
orf1235[b]	322	WP_019084814.1	100	P21908.2	81	Glucokinase [*Komagataeibacter europaeus*]; Glucokinase=Glucose kinase [*Zymomonas mobilis* subsp. *mobilis* ZM4 = ATCC 31821]
orf1499[b]	204	WP_010507182.1	100	Q00384.2	74	Ketohydroxyglutarate aldolase [*Komagataeibacter europaeus*]; KHG/KDPG aldolase/Includes: RecName=4-hydroxy-2-oxoglutarate aldolase=2-keto-4-hydroxyglutarate aldolase=KHG-aldolase
orf1500[b]	612	WP_010507177.1	100	P21909.2	77	Phosphogluconate dehydratase [*Komagataeibacter europaeus*]; Phosphogluconate dehydratase=6-phosphogluconate dehydratase [*Zymomonas mobilis* subsp. *mobilis* ZM4 = ATCC 31821]
orf1660	358	WP_026019783.1	100	–	–	Gluconolactonase [*Komagataeibacter europaeus*]
orf1676[b]	435	WP_019086743.1	100	O54068.2	74	UDP-glucose 6-dehydrogenase [*Komagataeibacter europaeus*]; UDP-glucose 6-dehydrogenase=UDP-Glc dehydrogenase=UDP-GlcDH= UDPGDH [*Sinorhizobium meliloti* 1021]
orf2085[b]	509	WP_019086471.1	100	P29686.2	65	Glucose 6-phosphate dehydrogenase [*Komagataeibacter europaeus*]; Glucose-6-phosphate 1-dehydrogenase=G6PD [*Synechococcus elongatus* PCC 7942]
orf2087[b]	952	WP_010508299.1	100	B8GMX9.1	67	Phosoglucose isomerase; Transaldolase [*Thioalkalivibrio sulfidiphilus* HL-EbGr7]
orf2251[b]	320	AHI26643.1	92	O84903.1	61	UDP-glucose 4-epimerase [*Gluconacetobacter xylinus* E25]; UDP-glucose 4-epimerase=Galactowaldenase=UDP-galactose 4-epimerase [*Lactobacillus casei*]
orf2600[b]	367	WP_019085359.1	100	–	–	Gluconolactonase [*Komagataeibacter europaeus*]
orf2602	253	WP_010510599.1	100	P52037.2	64	Glucose-1-dehydrogenase [*Komagataeibacter europaeus*]; Uncharacterized oxidoreductase YgfF [*Escherichia coli* K-12]
orf2611	373	WP_019091209.1	99	–	–	Gluconolactonase [*Komagataeibacter europaeus*]
orf2955[b]	497	WP_029335445.1	100	P29686.2	65	Glucose 6-phosphate dehydrogenase [*Komagataeibacter europaeus*]; Glucose-6-phosphate 1-dehydrogenase=G6PD [*Synechococcus elongatus* PCC 7942]
orf3032[b]	728	WP_019085328.1	100	Q70JN9.1	83	Glucose dehydrogenase [*Komagataeibacter europaeus*]; Glycerol

						dehydrogenase large subunit=D-arabitol dehydrogenase large subunit= ARDH=D-sorbitol dehydrogenase subunit
orf3082[b]	510	WP_010510226.1	100	P29686.2	65	Glucose-6-phosphate 1-dehydrogenase [*Komagataeibacter europaeus*]; Glucose-6-phosphate 1-dehydrogenase=G6PD [*Synechococcus elongatus* PCC 7942]
orf3085[b]	955	WP_019087003.1	100	P48993.2	67	Phosoglucose isomerase; Transaldolase 2 [*Nostoc* sp. PCC 7120]
orf3121[b]	438	WP_019086388.1	100	O54068.2	81	UDP-glucose 6-dehydrogenase [*Komagataeibacter europaeus*]; UDP-glucose 6-dehydrogenase=UDP-Glc dehydrogenase=UDP-GlcDH= UDPGDH [*Sinorhizobium meliloti* 1021]
orf3285[b]	183	WP_010510136.1	100	P46859.4	64	Carbohydrate kinase [*Komagataeibacter europaeus*]; Thermoresistant gluconokinase=Gluconate kinase 2 [*Escherichia coli* K-12]
**C Fructose metabolism (5/6)**						
orf0733[b]	328	WP_010507481.1	100	Q03224.1	73	Fructose 1,6-bisphosphatase [*Komagataeibacter europaeus*]; Fructose-1,6-bisphosphatase class 2=FBPase class 2=D-fructose-1,6-bisphosphate 1-phosphohydrolase class 2 [*Bacillus subtilis* subsp.]
orf0734[b]	368	WP_019091548.1	100	Q0PAS0.1	69	Fructose-bisphosphate aldolase [*Komagataeibacter europaeus*]; Fructose-bisphosphate aldolase=FBP aldolase=FBPA=Fructose-1,6-bisphosphate aldolase [*Campylobacter jejuni* subsp. *jejuni* N]
orf1224[b]	302	WP_026019736.1	100	P23917.2	66	Hypothetical protein [*Komagataeibacter europaeus*]; Fructokinase=D-fructose kinase=Manno(fructo)kinase [*Escherichia coli* K-12]
orf1727[b]	607	WP_010509784.1	100	Q5FUY5.3	81	Glucosamine-fructose-6-phosphate aminotransferase [*Komagataeibacter europaeus*]; Glutamine-fructose-6-phosphate aminotransferase [isomerizing]
orf2671	373	WP_010510015.1	100	Q07982.2	57	Glucose-fructose oxidoreductase [*Komagataeibacter europaeus*]; Glucose-fructose oxidoreductase=GFOR=Precursor [*Zymomonas mobilis* subsp. *mobilis* ZM4 = ATCC 31821]
orf3090[b]	293	WP_019087524.1	100	Q73QV3.1	71	Fructose-bisphosphate aldolase [*Komagataeibacter europaeus*]; Fructose-bisphosphate aldolase class 1=Fructose-bisphosphate

						aldolase class I=FBP aldolase [*Treponema denticola* ATCC 35405]
**D Mannitol/Mannose metabolism (10/10)**						
**Mannitol (1/1)**						
orf2102[b]	487	WP_010508282.1	100	P80354.2	72	Polyol:NADP oxidoreductase [*Komagataeibacter europaeus*]; Polyol:NADP oxidoreductase [*Gluconobacter oxydans* 621H]; Mannitol 2-dehydrogenase (String tophit description)[g]
**Mannose (9/9)**						
orf0913[b]	148	WP_010509528.1	100	–	–	PTS fructose transporter subunit IIA [*Komagataeibacter europaeus*]
orf1123[b]	469	WP_019084881.1	100	P45632.2	71	Phosphomannomutase [*Komagataeibacter europaeus*]; Phosphomannomutase=PMM
orf1464[b]	41	WP_010507232.1	100	–	–	Glycosyl transferase family 1 [*Komagataeibacter europaeus*]
orf1465[b]	394	WP_010507232.1	99	–	–	Glycosyl transferase family 1 [*Komagataeibacter europaeus*]
orf1678[b]	489	WP_019090783.1	100	P24174.3	65	Mannose-1-phosphate guanylyltransferase [*Komagataeibacter europaeus*]; Mannose-1-phosphate guanylyltransferase=GDP-mannose pyrophosphorylase=GMP=GMPP [*Escherichia coli* K-12]
orf2266[b]	376	WP_010508897.1	99	P29954.1	43	Mannose-6-phosphate isomerase [*Komagataeibacter europaeus*]; Mannose-6-phosphate isomerase=Phosphohexomutase=Phosphomannose isomerase=PMI [*Sinorhizobium meliloti* 1021]
orf2311[b]	249	WP_019091408.1	100	–	–	Mannose-1-phosphate guanylyltransferase [*Komagataeibacter europaeus*]
orf2787[b]	485	WP_010508523.1	100	P55356.1	62	Phosphomannomutase [*Komagataeibacter europaeus*]; Phosphomannomutase=PMM [*Sinorhizobium fredii* NGR234]
orf3168[b]	435	WP_010507285.1	99	B0RVK6.1	68	Mannose-1-phosphate guanylyltransferase [*Komagataeibacter europaeus*]; Xanthan biosynthesis protein XanB=Mannose-6-phosphate isomerase=Phosphohexomutase=Phosphomannomutase
**E Trehalase metabolism (6/6)**						

orf0830[b]	737	WP_010512067.1	89	B7LSZ0.1	65	Trehalase, partial [*Komagataeibacter europaeus*]; Cytoplasmic trehalase=Alpha,alpha-trehalase=Alpha,alpha-trehalose glucohydrolase [*Escherichia fergusonii* ATCC 35469]
orf1249[b]	459	WP_010507570.1	100	P55612.1	59	Alpha,alpha-trehalose-phosphate synthase [*Komagataeibacter europaeus*]; Probable alpha,alpha-trehalose-phosphate synthase [UDP-forming]
orf1859[b]	914	WP_019090831.1	99	P9WQ20.1	53	Malto-oligosyltrehalose synthase [Komagataeibacter europaeus]; Putative maltooligosyl trehalose synthase=(1,4)-alpha-D-glucan 1-alpha-D-glucosylmutase [*Mycobacterium tuberculosis* CDC1551]
orf1861[b]	593	WP_010508465.1	99	Q9RX51.1	60	Malto-oligosyltrehalose trehalohydrolase [*Komagataeibacter europaeus*]; Malto-oligosyltrehalose trehalohydrolase=MTHase=4-alpha-D-((1-)4)-alpha-D-glucano)trehalose trehalohydrolase
orf2434[b]	450	WP_010508835.1	100	Q2NTK9.1	68	Alpha,alpha-trehalose-phosphate synthase [*Komagataeibacter europaeus*]; Alpha,alpha-trehalose-phosphate synthase [UDP-forming]
orf2435[b]	251	WP_019091171.1	100	P31678.2	54	Trehalose-phosphatase [*Komagataeibacter europaeus*]; Trehalose-6-phosphate phosphatase=TPP=Osmoregulatory trehalose synthesis protein B=Trehalose 6-phosphate phosphatas
**F Glycogen/Starch metabolism (4/4)**						
orf1862[b]	704	WP_010508464.1	100	P0A4Y5.1	61	Glycogen debranching protein [*Komagataeibacter europaeus*]; Glycogen operon protein GlgX homolog [*Mycobacterium bovis* AF2122/97]
orf1863[b]	739	WP_010508463.1	100	Q8PE48.1	71	Glycogen branching protein [*Komagataeibacter europaeus*]; 1,4-alpha-glucan branching enzyme GlgB 1=1,4-alpha-D-glucan:1,4-alpha-D-glucan 6-glucosyl-transferase 1
orf1864[b]	540	WP_019090827.1	100	Q6NCT7.1	68	Glycogen synthase [*Komagataeibacter europaeus*]; Glycogen synthase= Starch [bacterial glycogen]
orf1865[b]	835	WP_019090826.1	99	Q9YGA7.1	59	Alpha-glucan phosphorylase [*Komagataeibacter europaeus*]; Maltodextrin phosphorylase [*Thermococcus litoralis* DSM 5473]

**G Acetate metabolism (12/16)**						
orf0455[b]	551	WP_026018392.1	99	P06672.1	50	Pyruvate decarboxylase [*Komagataeibacter europaeus*]; Pyruvate decarboxylase=PDC [*Zymomonas mobilis* subsp. *mobilis* ZM4 = ATCC 31821]
orf0635[b]	484	WP_010507363.1	100	H8ZPX2.1	69	Aldehyde dehydrogenase [*Komagataeibacter europaeus*]; 3-succinoylsemialdehyde-pyridine dehydrogenase=SAPD [*Pseudomonas* sp.]
orf0652	707	WP_010507386.1	100	–	–	Aldehyde dehydrogenase [*Komagataeibacter europaeus*]
orf0747[b]	503	WP_019086799.1	100	P37685.2	82	Aldehyde dehydrogenase [*Komagataeibacter europaeus*]; Aldehyde dehydrogenase B [*Escherichia coli* K-12]
orf0972[b]	399	WP_019084966.1	100	Q6N143.1	63	Acetate kinase [*Komagataeibacter europaeus*]; Acetate kinase=Acetokinase [*Rhodopseudomonas palustris* CGA009]
orf1155	773	WP_019091244.1	100	P17201.1	100	Aldehyde dehydrogenase [*Komagataeibacter europaeus*]; Membrane-bound aldehyde dehydrogenase [pyrroloquinoline-quinone]
orf1157	433	WP_019084855.1	100	Q88FX8.1	66	Aldehyde dehydrogenase [*Komagataeibacter europaeus*]; Nicotinate dehydrogenase subunit B=Nicotinate degradation protein B
orf1419[b]	345	WP_019084683.1	100	Q9X448.2	67	Phosphate acetyltransferase [*Komagataeibacter europaeus*]; Phosphate acetyltransferase=Phosphotransacetylase
orf1540[b]	482	WP_010507111.1	100	P25553.2	62	Aldehyde dehydrogenase [*Komagataeibacter europaeus*]; Lactaldehyde dehydrogenase=Aldehyde dehydrogenase A=Glycolaldehyde dehydrogenase [*Escherichia coli* K-12]
orf1658[b]	380	WP_019086891.1	99	Q59695.1	63	Branched-chain alpha-keto acid dehydrogenase subunit E2 [*Komagataeibacter europaeus*]; Dihydrolipoyllysine-residue acetyltransferase component of acetoin cleaving system=Acetoin dehydrogenase E2 component
orf2019[b]	564	WP_010509054.1	100	P06672.1	76	Pyruvate decarboxylase [*Komagataeibacter europaeus*]; Pyruvate decarboxylase=PDC [*Zymomonas mobilis* subsp. *mobilis* ZM4 = ATCC 31821]

orf2369[b]	505	WP_019091418.1	100	P38946.1	67	Acetyl-CoA hydrolase [*Komagataeibacter europaeus*]; Succinyl-CoA:coenzyme A transferase [*Clostridium kluyveri* DSM 555]
orf2878[b]	414	WP_019091078.1	100	P08461.3	64	Branched-chain alpha-keto acid dehydrogenase subunit E2 [*Komagataeibacter europaeus*]; Dihydrolipoyllysine-residue acetyltransferase component of pyruvate dehydrogenase complex, mitochondrial
orf3324	41	KDA05337.1	95	–	–	Aldehyde dehydrogenase [*Microbacterium* sp. CH12i]
orf3383[b]	581	WP_010515876.1	95	P28812.2	60	MULTISPECIES: acetyl-CoA synthetase [*Komagataeibacter*]; Uncharacterized protein PA3568 [*Pseudomonas aeruginosa* PAO1]
orf3493[b]	304	WP_019087399.1	99	Q9KWA3.1	57	Acetyl-CoA synthetase [*Komagataeibacter*]; Acetyl-coenzyme A synthetase=AcCoA synthetase=Acs=Acetate--CoA ligase=Acyl-activating enzyme
**H Glycerol metabolism (16/22)**						
orf0199[b]	308	WP_010506432.1	100	A9HS30.1	95	Phosphate acyltransferase [*Komagataeibacter europaeus*]; Phosphate acyltransferase=Acyl-ACP phosphotransacylase=Acyl-[acyl-carrier-protein]
orf0263[b]	251	WP_010506551.1	100	–	–	1-acyl-sn-glycerol-3-phosphate acyltransferase [*Komagataeibacter europaeus*]
orf0312[b]	517	WP_010506631.1	100	Q5FU33.1	80	Phosphoglyceromutase [*Komagataeibacter europaeus*]; 2,3-bisphosphoglycerate-independent phosphoglycerate mutase=BPG-independent PGAM=Phosphoglyceromutase=iPGM [*Gluconobacter oxydans*]
orf0690	296	WP_010507431.1	99	O31502.1	56	Diacylglycerol kinase [*Komagataeibacter europaeus*]; Diacylglycerol kinase=DAG kinase=DAGK [*Bacillus subtilis* subsp. *subtilis* str. 168]
orf0729	270	WP_019086028.1	100	Q51389.2	58	Glycerol transporter [*Komagataeibacter europaeus*]; Glycerol uptake facilitator protein=Glycerol diffusion facilitator [*Pseudomonas aeruginosa* PAO1]
orf0731[b]	520	WP_019086027.1	100	P52111.2	67	Glycerol-3-phosphate dehydrogenase [*Komagataeibacter europaeus*];

						Glycerol-3-phosphate dehydrogenase [*Pseudomonas aeruginosa* PAO1]
orf0732[b]	499	WP_010507480.1	100	Q5FP70.1	81	Glycerol kinase [*Komagataeibacter europaeus*]; Glycerol kinase=ATP:glycerol 3-phosphotransferase=Glycerokinase=GK [*Gluconobacter oxydans* 621H]
orf0735	252	WP_010507484.1	100	Q51391.2	69	DeoR faimly transcriptional regulator [*Komagataeibacter europaeus*]; Glycerol-3-phosphate regulon repressor [*Pseudomonas aeruginosa* PAO1]
orf0843	255	WP_019085631.1	100	–	–	Phosphoglycerate mutase [*Komagataeibacter europaeus*]
orf1078[b]	544	WP_019086540.1	100	P45510.3	60	Dihydroxyacetone kinase [*Komagataeibacter europaeus*]; Dihydroxyacetone kinase=DHA kinase=Glycerone kinase
orf1344[b]	323	WP_019090556.1	99	Q5FPS8.1	68	Glycerol-3-phosphate dehydrogenase [*Komagataeibacter europaeus*]; Glycerol-3-phosphate dehydrogenase [$NAD(P)^+$]
orf1526[b]	407	WP_010507127.1	100	Q0BVE2.1	84	Phosphoglycerate kinase [*Komagataeibacter europaeus*]; Phosphoglycerate kinase [*Granulibacter bethesdensis* CGDNIH1]
orf1527[b]	340	WP_010507126.1	100	P29272.1	74	Glyceraldehyde-3-phosphate dehydrogenase [*Komagataeibacter europaeus*]; Glyceraldehyde-3-phosphate dehydrogenase B; Short=GAPDH [*Rhodobacter sphaeroides*]
orf1774	326	WP_010509687.1	100	–	–	Diacylglycerol kinase [*Komagataeibacter europaeus*]
orf1877[b]	244	WP_019090822.1	99	Q8ZA34.1	58	CDP-diacylglycerol pyrophosphatase [*Komagataeibacter europaeus*]; CDP-diacylglycerol pyrophosphatase=CDP-diacylglycerol phosphatidylhydrolase=CDP-diglyceride hydrolase [*Yersinia pestis*]
orf1978[b]	212	WP_019084975.1	100	Q5FRH0.1	71	Glycerol-3-phosphate acyltransferase [*Komagataeibacter europaeus*]; Glycerol-3-phosphate acyltransferase=Acyl-PO4 G3P acyltransferase= Acyl-phosphate-glycerol-3-phosphate acyltransferase
orf2262[b]	376	WP_019091785.1	100	–	–	1-acyl-sn-glycerol-3-phosphate acyltransferase [*Komagataeibacter europaeus*]
orf2355[b]	199	WP_010508023.1	100	Q68XS5.1	65	CDP-diacylglycerol--glycerol-3-phosphate 3-phosphatidyltransferase [*Komagataeibacter europaeus*]; CDP-diacylglycerol--glycerol-3-

						phosphate 3-phosphatidyltransferase [*Rickettsia typhi* str. Wilmington]
orf2408[b]	196	WP_019086086.1	99	O07617.1	76	Phosphoglycerate mutase [*Komagataeibacter europaeus*]; Uncharacterized phosphatase PhoE [*Bacillus subtilis* subsp. *subtilis* str. 168]
orf2923	176	WP_010508211.1	99	–	–	Phosphoglycerate mutase [*Komagataeibacter europaeus*]
orf3031[b]	124	WP_019085329.1	100	Q8L1D5.3	75	Glycerol dehydrogenase [*Komagataeibacter europaeus*]; Glycerol dehydrogenase small subunit=D-arabitol dehydrogenase small subunit= ARDH=D-sorbitol dehydrogenase subunit
orf3498[b]	465	WP_025811963.1	98	Q606T2.1	81	Enolase [*Komagataeibacter kakiaceti*]; Enolase 1=2-phospho-D-glycerate hydro-lyase 1=2-phosphoglycerate dehydratase 1 [*Methylococcus capsulatus* str. Bath]
**I Alcohol metabolism (10/27)**						
orf0432	444	WP_019084594.1	99	O34215.1	65	Alcohol dehydrogenase [*Komagataeibacter europaeus*]; Gluconate 2-dehydrogenase cytochrome c subunit=GA 2-DH cytochrome c subunit=GADH cytochrome c subunit/Flags: Precursor [*Pantoea cypripedii*] (*Pectobacterium cypripedii*) (*Erwinia cypripedii*)
orf0533	353	WP_019091327.1	99	P39346.1	59	Zinc-binding alcohol dehydrogenase [*Komagataeibacter europaeus*]; L-idonate 5-dehydrogenase (NAD(P)($^{+}$)) [*Escherichia coli* K-12]
orf0537	140	WP_010509603.1	100	–	–	Alcohol dehydrogenase [*Komagataeibacter europaeus*]
orf0599[b]	718	WP_010507316.1	99		78	Alcohol dehydrogenase [*Komagataeibacter europaeus*]; Alcohol dehydrogenase [cytochrome c]
orf0606[b]	340	WP_026019629.1	99	Q5SKS4.1	57	Alcohol dehydrogenase [*Komagataeibacter europaeus*]; L-threonine 3-dehydrogenase [*Thermus thermophilus* HB8]
orf0620	538	WP_019086999.1	100	Q9WWW2.1	60	Alcohol dehydrogenase [*Komagataeibacter europaeus*]; Alcohol dehydrogenase [acceptor]
orf0653	416	WP_019091808.1	99	M1V1V5.1	56	Alcohol dehydrogenase [*Komagataeibacter europaeus*]; Methylamine dehydrogenase light chain=MADH=Methylamine dehydrogenase

						(amicyanin)/Flags: Precursor
orf1317[b]	708	WP_026019545.1	100	Q44002.1	100	Alcohol dehydrogenase [*Komagataeibacter europaeus*]; Alcohol dehydrogenase [cytochrome c]
orf1318	473	WP_026019546.1	100	P0A388.1	100	Alcohol dehydrogenase [*Komagataeibacter europaeus*]; Alcohol dehydrogenase cytochrome c subunit/Flags: Precursor CYCA_KOMEU RecName=Alcohol dehydrogenase cytochrome c subunit
orf2233[b]	700	WP_029335389.1	100	Q44002.1	81	Alcohol dehydrogenase [*Komagataeibacter europaeus*]; Alcohol dehydrogenase [cytochrome c]
orf2385[b]	395	WP_010508057.1	100	P45382.1	82	Alcohol dehydrogenase [*Komagataeibacter europaeus*]; S-(hydroxymethyl)glutathione dehydrogenase=Glutathione-dependent formaldehyde dehydrogenase=FALDH=FDH=GSH-FDH Precursor [*Pantoea cypripedii*]
orf2443[b]	325	WP_026019657.1	100	P9WQC0.1	58	Alcohol dehydrogenase [*Komagataeibacter europaeus*]; Probable alcohol dehydrogenase AdhA [*Mycobacterium tuberculosis* CDC1551]
orf2591	148	WP_010511043.1	100	–	–	Alcohol dehydrogenase [*Komagataeibacter europaeus*]
orf2645[b]	378	WP_010509977.1	100	Q9HTE3.1	80	Aldehyde dismutase [*Komagataeibacter europaeus*]; Glutathione-independent formaldehyde dehydrogenase=FALDH=FDH [*Pseudomonas aeruginosa* PAO1]; Alcohol dehydrogenase (String tophit description)
orf2727	350	WP_010510466.1	100	P76113.3	77	2-alkenal reductase [*Komagataeibacter europaeus*]; NADPH-dependent curcumin reductase; AltName: Full=NADPH-dependent curcumin/dihydrocurcumin reductase [*Escherichia coli* K-12]; Alcohol dehydrogenase (String tophit description)
orf2753	409	WP_019091387.1	100	Q88FX8.1	52	Alcohol dehydrogenase [*Komagataeibacter europaeus*]; Nicotinate dehydrogenase subunit B=Nicotinate degradation protein B=Nicotinate dehydrogenase large subunit
orf2908[b]	340	WP_010507980.1	100	P42327.1	73	Alcohol dehydrogenase [*Komagataeibacter europaeus*]; Alcohol dehydrogenase=ADH

orf2920[b]	326	WP_010508213.1	100	Q3J6K9.1	70	Alcohol dehydrogenase [*Komagataeibacter europaeus*]; Acrylyl-CoA reductase AcuI=Acryloyl-coenzyme A reductase [*Rhodobacter sphaeroides* 2.4.1]
orf2999	340	WP_008852694.1	98	O94564.1	61	NADPH:quinone oxidoreductase [*Gluconobacter morbifer*]; Zinc-type alcohol dehydrogenase-like protein C1773.06c [*Schizosaccharomyces pombe* 972h-]
orf3016	67	WP_014229973.1	81	–	–	Alcohol dehydrogenase [*Klebsiella oxytoca*]
orf3019	293	WP_029606267.1	96	–	–	Alcohol dehydrogenase [*Kozakia baliensis*]
orf3194	161	WP_019086363.1	99	–	–	Alcohol dehydrogenase [*Komagataeibacter europaeus*]
orf3322	291	EXU69213.1	66	O94564.1	54	Alcohol dehydrogenase [*Streptomyces* sp. PRh5]; Zinc-type alcohol dehydrogenase-like protein C1773.06c [*Schizosaccharomyces pombe* 972h-]
orf3394[b]	371	AHI26390.1	97	O94564.1	54	Alcohol dehydrogenase zinc-binding domain protein [*Gluconacetobacter xylinus* E25]; Zinc-type alcohol dehydrogenase-like protein C1773.06c [*Schizosaccharomyces pombe* 972h-]
orf3410	163	WP_007400709.1	100	Q49WS9.1	60	Oxidoreductase [*Gluconacetobacter* sp. SXCC-1]; Uncharacterized oxidoreductase SSP1627 [*Staphylococcus saprophyticus* subsp. *saprophyticus* ATCC 15305]; Short chain alcohol dehydrogenase-related dehydrogenase (String tophit description)
orf3438	332	WP_010510265.1	99	O94564.1	57	NADPH:quinone oxidoreductase [*Komagataeibacter europaeus*]; Zinc-type alcohol dehydrogenase-like protein C1773.06c [*Schizosaccharomyces pombe* 972h-]; Alcohol dehydrogenase, zinc-containing (String tophit description)
orf3606	306	WP_003631306.1	98	P76113.3	63	Alcohol dehydrogenase [*Acetobacter pasteurianus*]; NADPH-dependent curcumin reductasel=NADPH-dependent curcumin/dihydrocurcumin reductase [*Escherichia coli* K-12]
**J Lactate metabolism (9/15)**						
orf0291	485	WP_010506596.1	99	P46681.1	57	D-2-hydroxyacid dehydrogenase [*Komagataeibacter europaeus*]; D-

						lactate dehydrogenase [cytochrome]
orf0624[b]	380	WP_010507348.1	100	A8HTC9.1	57	Oxidoreductase [*Komagataeibacter europaeus*]; L-lactate dehydrogenase [*Azorhizobium caulinodans* ORS 571]
orf0627[b]	451	WP_010507354.1	100	Q7TNG8.1	63	2-hydroxy-acid oxidase [*Komagataeibacter europaeus*]; Probable D-lactate dehydrogenase, mitochondrial=DLD=Lactate dehydrogenase D/Flags: Precursor [*Mus musculus*]
orf1005[b]	606	WP_010510065.1	100	P40811.3	66	Acetolactate synthase [*Komagataeibacter europaeus*]; Acetolactate synthase isozyme 3 large subunit=HAS-III=ALS-III=Acetohydroxy-acid synthase III large subunit
orf1006[b]	186	BAP16206.1	100	P21622.2	65	Acetohydroxyacid synthase isozyme III small regulatory subunit [*Komagataeibacter europaeus*]; Acetolactate synthase isozyme 3 small subunit=ALS-III=Acetohydroxy-acid synthase III small subunit=AHAS-III
orf1232[b]	566	WP_019091765.1	100	P20906.2	63	Acetolactate synthase [*Komagataeibacter europaeus*]; Benzoylformate decarboxylase=BFD=BFDC [*Pseudomonas putida*]
orf2015[b]	261	BAO00818.1	100	P05361.1	61	Alpha-acetolactate decarboxylase [*Komagataeibacter europaeus*]; Alpha-acetolactate decarboxylase
orf2016[b]	548	WP_010509049.1	100	P27696.1	73	Acetolactate synthase [*Komagataeibacter europaeus*]; Acetolactate synthase, catabolic=ALS [*Klebsiella pneumoniae*]
orf2018[b]	577	WP_029335418.1	100	P06149.3	77	Lactate dehydrogenase [*Komagataeibacter europaeus*]; D-lactate dehydrogenase=Respiratory D-lactate dehydrogenase [*Escherichia coli* K-12]
orf2243	268	WP_010508744.1	99	P31777.1	56	Lactate dehydrogenase [*Komagataeibacter europaeus*]; Ribosomal RNA large subunit methyltransferase J=23S rRNA (adenine(2030)-N6)-methyltransferase=23S rRNA m6A2030 methyltransferase
orf2426	247	WP_010508820.1	100	Q9KBU2.1	65	Fe-S oxidoreductase [*Komagataeibacter europaeus*]; Lactate utilization protein A [*Bacillus halodurans* C-125]
orf2427	470	WP_010508822.1	100	A7GMJ3.1	58	4Fe-4S ferredoxin [*Komagataeibacter europaeus*]; Lactate utilization protein B [*Bacillus cytotoxicus* NVH 391-98]

orf3211[b]	430	WP_010507526.1	100	Q7TNG8.1	65	2-hydroxy-acid oxidase [*Komagataeibacter europaeus*]; Probable D-lactate dehydrogenase, mitochondrial= DLD=Lactate dehydrogenase D/Flags: Precursor [*Mus musculus*]
orf3653	277	WP_007400689.1	100	–	–	Lactate dehydrogenase [*Gluconacetobacter* sp. SXCC-1]
orf3654	71	WP_014106764.1	100	–	–	Lactate dehydrogenase [*Komagataeibacter medellinensis*]
**K Others[e] (13/13)**						
orf0973[b]	789	WP_010511491.1	100	Q8YTZ6.1	82	Phosphoketolase [*Komagataeibacter europaeus*]; Probable phosphoketolase 2 [*Nostoc* sp. PCC 7120]
orf1528[b]	623	WP_019091339.1	100	P56900.2	62	Transketolase [*Komagataeibacter europaeus*]; Transketolase=TK [*Sinorhizobium medicae* WSM419]
orf2086[b]	332	WP_019090790.1	100	O83351.1	56	6-phosphogluconate dehydrogenase [*Komagataeibacter europaeus*]; 6-phosphogluconate dehydrogenase, decarboxylating [*Treponema pallidum* subsp. *pallidum* str. Nichols]
orf2088[b]	688	WP_026018728.1	100	Q9KAD7.1	70	Transketolase [*Komagataeibacter europaeus*]; Transketolase=TK [*Bacillus halodurans* C-125]
orf2244[b]	317	WP_019087370.1	99	P0A9J7.1	52	Ribokinase [*Komagataeibacter europaeus*]; Ribokinase [*Escherichia coli* O157:H7]
orf2409[b]	329	WP_010508788.1	100	Q0BPP0.1	90	Ribose-phosphate pyrophosphokinase [*Komagataeibacter europaeus*]; Ribose-phosphate pyrophosphokinase=RPPK=Phosphoribosyl pyrophosphate synthase=P-Rib-PP synthase
orf2867[b]	286	WP_019091083.1	100	A9HJ86.1	81	Triosephosphate isomerase [*Komagataeibacter europaeus*]; Triosephosphate isomerase=TIM=Triose-phosphate isomerase [*Gluconacetobacter diazotrophicus* PA1 5]
orf3083[b]	328	WP_019087002.1	100	P80859.4	56	6-phosphogluconate dehydrogenase [*Komagataeibacter europaeus*]; 6-phosphogluconate dehydrogenase, NADP($^+$)-dependent, decarboxylating=GNTZII [*Bacillus subtilis* subsp. *subtilis* str. 168]
orf3086[b]	672	WP_010510230.1	100	Q9KAD7.1	70	Transketolase [*Komagataeibacter europaeus*]; Transketolase=TK [*Bacillus halodurans* C-125]
orf3283[b]	110	WP_010512193.1	100	P74618.3	59	6-phosphogluconolactonase [*Komagataeibacter europaeus*]; 6-

						phosphogluconolactonase=6PGL [*Synechocystis* sp. PCC 6803 substr. *Kazusa*]
orf3284[b]	234	WP_010510134.1	100	Q5FQ98.1	76	Ribose 5-phosphate isomerase [*Komagataeibacter europaeus*]; Ribose-5-phosphate isomerase A=Phosphoriboisomerase A=PRI [*Gluconobacter oxydans* 621H]
orf3467[b]	165	WP_019092434.1	100	P74618.3	54	6-phosphogluconolactonase, partial [Komagataeibacter europaeus]; 6-phosphogluconolactonase=6PGL [*Synechocystis* sp. PCC 6803 substr. *Kazusa*]
orf3468[b]	247	WP_026018709.1	100	Q5FQ98.1	70	Ribose 5-phosphate isomerase [*Komagataeibacter europaeus*]; Ribose-5-phosphate isomerase A=Phosphoriboisomerase A=PRI [*Gluconobacter oxydans* 621H]

[a] The numbers indicate the orfs that can be annotated to Kyoto Encyclopedia of Genes and Genomes (KEGG) pathways out of total orfs (Table S3). [b] The orfs that can be annotated to known pathways in KEGG pathway database as shown in Table S3. [c, d] The full sets of cellulose synthase genes in the *bcs2* and *bcs1* operons, respectively. [e] Include the metabolic intermediates between glucose and glycerol or fructose and glycerol. [f] No annotation is available. [g] Although the results of Nr top hit and (or) Swissprot top hit cannot be obtained, the String tophit description can annotate the certain orfs to the corresponding proteins.

Table S2. Open reading frames and corresponding proteins information based on KEGG pathway annotation.

Open reading frames ID	Enzyme ID	Enzyme names	Ko ID (Gene ID)	Ko names (Gene names)	KEGG ID
**A Cellulose synthesis**					
**A-1 Synthesis**					
orf0140; orf0495; orf1578; orf1579	2.4.1.12	Cellulose synthase (UDP-forming)	K00694	*bcsA*	ko00500; ko01100
**A-2 Regulation**					
orf1575; orf1669	3.2.1.4	Endoglucanase	K01179	*cmcax*	ko00500; ko01100
orf1582	3.2.1.21	Beta-glucosidase	K05349	*bglx*	ko00460; ko00500; ko00940; ko01100; ko01110
**B Glucose metabolism**					
orf0040; orf2085; orf2955; orf3082	1.1.1.49/ 1.1.1.363	Glucose-6-phosphate 1-dehydrogenase	K00036	*G6PD*; *zwf*	ko00030; ko00480; ko01100; ko01110; ko01120; ko01200
orf0238; orf0534; orf3285	2.7.1.12	Gluconokinase=Gluconate kinase	K00851	*gntK*; *idnK*	ko00030; ko01100; ko01110; ko01120; ko01200
orf1086	5.4.2.2	Phosphoglucomutase	K01835	*pgm*	ko00010; ko00030; ko00052; ko00230; ko00500; ko00520; ko00521; ko01100; ko01110; ko01120
orf1122	2.7.7.9	UTP--glucose-1-phosphate uridylyltransferase	K00963	*UGP2*; *galU*; *galF*	ko00040; ko00052; ko00500; ko00520; ko01100; ko01110
orf1159; orf3032	1.1.5.2	Guinoprotein glucose dehydrogenase	K00117	*gcd*	ko00030; ko01100; ko01110
orf1235	2.7.1.2	Glucokinase	K00845	*GCK*; *glk*	ko00010; ko00052; ko00500; ko00520; ko00521; ko00524; ko01100; ko01110; ko01120; ko01200
orf1499	4.1.2.14/ 4.1.3.42	2-dehydro-3-deoxyphosphogluconate aldolase /	K01625	*eda*	ko00030; ko00630; ko01100; ko01120; ko01200

		(4S)-4-hydroxy-2-oxoglutarate aldolase= Ketohydroxyglutarate aldolase			
orf1500	4.2.1.12	Phosphogluconate dehydratase	K01690	*edd*	ko00030; ko01100; ko01120; ko01200
orf1676; orf3121	1.1.1.22	UDP-glucose 6-dehydrogenase	K00012	*UGDH*; *ugd*	ko00040; ko00053; ko00500; ko00520; ko01100; ko01110
orf2087; orf3085	5.3.1.9/ 2.2.1.2	Glucose-6-phosphate isomerase= Phosoglucose isomerase/Transaldolase	K13810	*tal-pgi*	ko00010; ko00030; ko00500; ko00520; ko01100; ko01110; ko01120; ko01200; ko01230
orf2251	5.1.3.2	UDP-glucose 4-epimerase=Galactowaldenase	K01784	*galE*; *GALE*	ko00052; ko00520; ko01100; ko01110
orf2600	3.1.1.17	Gluconolactonase	K01053	*gnl*; *RGN*	ko00030; ko00053; ko00930; ko01100; ko01110; ko01120; ko01200; ko01220
**C Fructose metabolism**					
orf0733	3.1.3.11/ 3.1.3.37	Fructose 1,6-bisphosphatase II/ sedoheptulose-1,7-bisphosphatase	K11532	*glpX-SEBP*	ko00010; ko00030; ko00051; ko00680; ko00710; ko01100; ko01110; ko01120; ko01200
orf0734	4.1.2.13	Fructose-bisphosphate aldolase, class II	K01624	*FBA*; *fbaA*	ko00010; ko00030; ko00051; ko00680; ko00710; ko01100; ko01110; ko01120; ko01200; ko01230
orf1224	2.7.1.4	Fructokinase	K00847	*scrK*	ko00051; ko00500; ko00520; ko01100
orf1727	2.6.1.16	Glucosamine--fructose-6-phosphate aminotransferase (isomerizing)	K00820	*glmS*; *GFPT*	ko00250; ko00520; ko01100; ko01110
orf3090	4.1.2.13	Fructose-bisphosphate aldolase, class I	K01623	*ALDO*	ko00010; ko00030; ko00051; ko00680; ko00710; ko01100; ko01110; ko01120; ko01200; ko01230
**D Mannitol/Mannose metabolism**					

**D-1 Mannitol**					
orf2102	1.1.1.67	Mannitol 2-dehydrogenase	K00045	*mtlK*	ko00051
**D-2 Mannose**					
orf0913	2.7.1.191[a]	PTS system, mannose-specific IIA component	K02793	*PTS-Man-EIIA*; *manX*	ko00051; ko00520; ko01100; ko02060
orf1123; orf2787	5.4.2.8	Phosphomannomutase	K01840	*manB*	ko00051; ko00520; ko01100; ko01110
orf1464; orf1465	2.4.1.-	L-malate glycosyltransferase	K00754	*bshA*	ko00051
orf1678	2.7.7.13	Mannose-1-phosphate guanylyltransferase	K16011	*algA*; *xanB*; *rfbA*; *wbpW*; *pslB*	ko00051; ko00520; ko01100; ko01110
orf2266	5.3.1.8	Mannose-6-phosphate isomerase	K01809	*manA*	ko00051; ko00520; ko01100; ko01110
orf2311	2.7.7.13	Mannose-1-phosphate guanylyltransferase	K00966	*GMPP*	ko00051; ko00520; ko01100; ko01110
orf3168	2.7.7.13	Mannose-1-phosphate guanylyltransferase	K00971	*manC*; *cpsB*	ko00051; ko00520; ko01100; ko01110
**E Trehalase metabolism**					
orf0830	3.2.1.28	Alpha,alpha-trehalase	K01194	*TREH*; *treA/F*	ko00500; ko01100
orf1249; orf2434	2.4.1.15/ 2.4.1.347	Trehalose 6-phosphate synthase	K00697	*otsA*	ko00500; ko01100
orf1859	5.4.99.15	(1->4)-alpha-D-glucan 1-alpha-D-glucosylmutase	K06044	*treY*; *glgY*	ko00500; ko01100; Ko01110
orf1861	3.2.1.141	Maltooligosyltrehalose trehalohydrolase	K01236	*treZ*; *glgZ*	ko00500; ko01100; Ko01110
orf2435	3.1.3.12	Trehalose 6-phosphate phosphatase	K01087	*ostB*	ko00500; ko01100
**F Glycogen/Starch**					
orf1862	3.2.1.196	Glycogen debranching enzyme	K02438	*treX*; *glgX*	ko00500; ko01100; ko01110
orf1863	2.4.1.18	1,4-alpha-glucan branching enzyme	K00700	*GBE1*; *glgB*	ko00500; ko01100; ko01110
orf1864	2.4.1.21	Starch synthase	K00703	*glgA*	ko00500; ko01100; ko01110

orf1865	2.4.1.1	Glycogen phosphorylase	K00688	*glgP*; *PYG*	ko00500; ko01100; ko01110; ko04910
**G Acetate metabolism**					
orf0455; orf2019	4.1.1.74	Indolepyruvate decarboxylase	K04103	*ipdC*	ko00380; ko01100
orf0635; orf0747	1.2.1.3	aldehyde dehydrogenase (NAD^+)	K00128	*ALDH*	ko00010; ko00040; ko00053; ko00071; ko00280; ko00310; ko00330; ko00340; ko00380; ko00410; ko00561; ko00620; ko00625; ko00903; ko01100; ko01110; ko01120
orf0972	2.7.2.1	Acetate kinase	K00925	*ackA*	ko00430; ko00620; ko00640; ko00680; ko00720; ko01100; ko01120; ko01200
orf1419	2.3.1.8	Phosphate acetyltransferase	K00625	*pta*	ko00430; ko00620; ko00640; ko00680; ko00720; ko01100; ko01120; ko01200
orf1540	1.2.1.22/ 1.2.1.21	Lactaldehyde dehydrogenase/ glycolaldehyde dehydrogenase	K07248	*aldA*	ko00620; ko00630; ko01120
orf1658; orf2878	2.3.1.12	Pyruvate dehydrogenase E2 component (dihydrolipoamide acetyltransferase)	K00627	*DLAT*; *aceF*; *pdhC*	ko00010; ko00020; ko00620; ko01100; ko01110; ko01120; ko01200
orf2369	3.1.2.1	Acetyl-CoA hydrolase	K01067	*ACH1*	ko00620
orf3383	6.2.1.17	Propionyl-CoA synthetase	K01908	*prpE*	ko00640; ko01100
orf3493	6.2.1.1	Acetyl-CoA synthetase=Acetate--CoA ligase=Acyl-activating enzyme	K01895	*ACSS*; *acs*	ko00010; ko00620; ko00640; ko00680; ko00720; ko01100; ko01110; ko01120; ko01200
**H Glycerol metabolism**					
orf0199	2.3.1.15	Glycerol-3-phosphate acyltransferase PlsX	K03621	*plsX*	ko00561; ko00564; ko01100
orf0263; orf2262	2.3.1.51	1-acyl-sn-glycerol-3-phosphate acyltransferase	K00655	*plsC*	ko00561; ko00564; ko01100
orf0312	5.4.2.12	2,3-bisphosphoglycerate-independent phosphoglycerate	K15633	*gpmI*	ko00010; ko00260; ko00680; ko01100; ko01110; ko01120; ko01200; ko01230

		mutase			
orf0731	1.1.5.3	Glycerol-3-phosphate dehydrogenase	K00111	*glpA/D*	ko00564
orf0732	2.7.1.30	Glycerol kinase	K00864	*glpK*; *GK*	ko00561; ko01100; ko03320; ko04626
orf1078	2.7.1.28/ 2.7.1.29/ 4.6.1.15	Triose/dihydroxyacetone kinase / FAD-AMP lyase (cyclizing)	K00863	*DAK1/2*; *TKFC*	ko00561; ko00680; ko01100; ko01120; ko01200; ko04622
orf1344	1.1.1.94	Glycerol-3-phosphate dehydrogenase [NAD(P)$^+$]	K00057	*gpsA*	ko00564
orf1526	2.7.2.3	Phosphoglycerate kinase	K00927	*PGK*; *pgk*	ko00010; ko00710; ko01100; ko01110; ko01120; ko01200; ko01230
orf1527	1.2.1.12	Glyceraldehyde 3-phosphate dehydrogenase	K00134	*GAPDH*; *gapA*	ko00010; ko00710; ko01100; ko01110; ko01120; ko01200; ko01230; ko04066; ko05010
orf1877	3.6.1.26	CDP-diacylglycerol pyrophosphatase	K01521	*cdh*	ko00564
orf1978	2.3.1.15	Glycerol-3-phosphate acyltransferase PlsY	K08591	*plsY*	ko00561; ko00564; ko01100
orf2355	2.7.8.5	CDP-diacylglycerol--glycerol-3-phosphate 3-phosphatidyltransferase	K00995	*pgsA*; *PGS1*	ko00564; ko01100
orf2408	5.4.2.12	Probable phosphoglycerate mutase	K15634	*gpmB*	ko00010; ko00260; ko00680; ko01100; ko01110; ko01120; ko01200; ko01230
orf3031	1.1.5.2	Quinoprotein glucose dehydrogenase (Glycerol dehydrogenase)[b]	K00117	*gcd*	ko00030; ko01100; ko01110
orf3498	4.2.1.11	Enolase	K01689	*eno*; *ENO*	ko00010; ko00680; ko01100; ko01110; ko01120; ko01200; ko01230; ko03018; ko04066

**I Alcohol metabolism**					
orf0599; orf1317; orf2233	1.1.1.2.8	Alcohol dehydrogenase (cytochrome c)	K00114	*exaA*	ko00010; ko00625; ko01100; ko01110; ko01120
orf0606	1.1.1.4/ 1.1.1.303	(R,R)-butanediol dehydrogenase/meso-butanediol dehydrogenase/diacetyl reductase	K00004	*BDH*; *butB*	ko00650
orf2385	1.1.1.284/ 1.1.1.1	S-(hydroxymethyl)glutathione dehydrogenase/alcohol dehydrogenase	K00121	*frmA*; *ADH5*; *adhC*	ko00010; ko00071; ko00350; ko00625; ko00626; ko00680; ko00830; ko00980; ko00982; ko01100; ko01110; ko01120; ko01200; ko01220; ko05204
orf2443; orf2908	1.1.1.1	Alcohol dehydrogenase, propanol-preferring	K13953	*adhP*	ko00010; ko00071; ko00350; ko00625; ko00626; ko00830; ko00980; ko00982; ko01100; ko01110; ko01120; ko01220
orf2645	1.2.1.46	Glutathione-independent formaldehyde dehydrogenase	K00148	*fdhA*	ko00625; ko00680; ko01100; ko01120; ko01200
orf2920; orf3394	1.1.1.1	Alcohol dehydrogenase	K00001	*adh*	ko00010; ko00071; ko00350; ko00625; ko00626; ko00830; ko00980; ko00982; ko01100; ko01110; ko01120; ko01220
**J Lactate metabolism**					
orf0624	1.1.1.-	Butanol dehydrogenase	K00100	*bdhAB*	ko00051; ko00363; ko00591; ko00625; ko00650; ko01100; ko01120
orf0627; orf3211	1.1.2.4	D-lactate dehydrogenase (cytochrome)	K00102	*dld*; *LDHD*	ko00620
orf1005; orf2016	2.2.1.6L	Acetolactate synthase I/II/III large subunit	K01652	*ilvB*; *ilvG*; *ilvI*	ko00290; ko00650; ko00660; ko00770; ko01100; ko01110; ko01210; ko01230
orf1006	2.2.1.6S	Acetolactate synthase I/III small subunit	K01653	*ilvH*; *ilvN*	ko00290; ko00650; ko00660; ko00770; ko01100; ko01110; ko01210; ko01230
orf1232	4.1.1.7	Benzoylformate decarboxylase	K01576	*mdlC*	ko00627; ko01120
orf2015	4.1.1.5	Acetolactate decarboxylase	K01575	*alsD*; *budA*; *aldC*	ko00650; ko00660

orf2018	1.1.5.12	D-Lactate dehydrogenase (cytochrome)	K03777	*dld*	ko00620; ko01120
**K Others**[c]					
orf0973	4.1.2.9/ 4.1.2.22	Xylulose-5-phosphate/fructose-6-phosphate phosphoketolase	K01621	*xfp*; *xpk*	ko00030; ko00710; ko01100
orf1528; orf2088; orf3086	2.2.1.1	Transketolase	K00615	*tktA*/*B*	ko03440
orf2086; orf3083	1.1.1.44/ 1.1.1.343	6-phosphogluconate dehydrogenase	K00033	*PGD*; *gnd*; *gntZ*	ko00030; ko00480; ko01100; ko01110; ko01120; ko01200
orf2244	2.7.1.15	Ribokinase	K00852	*rbsK*; *RBKS*	ko00030
orf2409	2.7.6.1	Ribose-phosphate pyrophosphokinase	K00948	*PRPS*; *prsA*	ko00030; ko00230; ko01100; ko01110; ko01120; ko01200; ko01230
orf2867	5.3.1.1	Triosephosphate isomerase (TIM)	K01803	*TPI*; *tpiA*	ko00010; ko00051; ko00562; ko00710; ko01100; ko01110; ko01120; ko01200; ko01230
orf3283; orf3467	3.1.1.31	6-phosphogluconolactonase	K01057	*PGLS*; *pgl*; *devB*	ko00030; ko01100; ko01110; ko01120; ko01200
orf3284; orf3468	5.3.1.6	Ribose 5-phosphate isomerase	K01807	*rpiA*	ko00030; ko00710; ko01100; ko01110; ko01120; ko01200; ko01230

[a] The enzyme number 2.7.1.69 previously referred to permeases without a clear classification, and now it is divided into five groups including 2.7.1.191 (Protein-N(pi)-phosphohistidine--D-mannose phosphotransferase=Mannose PTS permease), 2.7.1.192 (Protein-N(pi)-phosphohistidine--N-acetylmuramate phosphotransferase=N-acetylmuramic acid PTS permease), 2.7.1.193 (Protein-N(pi)-phosphohistidine--N-acetyl-D-glucosamine phosphotransferase=N-acetyl-D-glucosamine PTS permease), 2.7.1.194 (Protein-N(pi)-phosphohistidine--L-ascorbate phosphotransferase=L-ascorbate PTS permease) and 2.7.1.195 (Protein-N(pi)-phosphohistidine--2-O-alpha-mannosyl-D-glycerate phosphotransferase=2-O-alpha-mannosyl-D-glycerate PTS permease). [b] Although the Nr top hit indicates that the orf3031 encodes glycerol dehydrogenase, the KEGG pathway analysis annotates it to quinoprotein glucose dehydrogenase, similar to orf1159 and orf3032. Here we assign it to glycerol metabolism. [c] Include the metabolic intermediates during the transformation between glucose and glycerol or fructose and glycerol.
